# Synthesis and biological evaluation of new derivatives of thieno-thiazole and dihydrothiazolo-thiazole scaffolds integrated with a pyrazoline nucleus as anticancer and multi-targeting kinase inhibitors†

**DOI:** 10.1039/d1ra08055e

**Published:** 2021-12-22

**Authors:** Ismail M. M. Othman, Zahra M. Alamshany, Nada Y. Tashkandi, Mohamed A. M. Gad-Elkareem, Somaia S. Abd El-Karim, Eman S. Nossier

**Affiliations:** Department of Chemistry, Faculty of Science, Al-Azhar University Assiut 71524 Egypt; Department of Chemistry, Faculty of Science, King Abdulaziz University P.O. Box 42805 Jeddah 21551 Saudi Arabia; Department of Therapeutic Chemistry, National Research Centre Dokki Cairo 12622 Egypt somaia_elkarim@hotmail.com; Department of Pharmaceutical Medicinal Chemistry, Faculty of Pharmacy (Girls), Al-Azhar University Cairo 11754 Egypt dremannossier@azhar.edu.eg

## Abstract

Deregulation of various protein kinases is considered as one of the important factors resulting in cancer development and metastasis, thus multi-targeting the kinase family is one of the most important strategies in current cancer therapy. This context represents the design and synthesis of two sets of derivatives bearing a pyrazoline-3-one ring conjugated either with a thieno[3,2-*d*]thiazole or with a dihydrothiazolo[4,5-*d*]thiazole scaffold *via* an NH linker, 3a–d and 5a–d respectively, using the pyrazolinone–thiazolinone derivative 1 as a key precursor. All the newly synthesized compounds were assessed *in vitro* for their anticancer activity against two cancer cell lines (MCF-7 and HepG-2). The safety profile of the most active cytotoxic candidates 1 and 3c was further examined against the normal cell line WI-38. The compounds 1 and 3c were further evaluated as multi-targeting kinase inhibitors against EGFR, VEGFR-2 and BRAF^V600E^, exhibiting promising suppression impact. Additionally, the latter compounds were investigated for their impact on cell cycle and apoptosis induction potential in the MCF-7 cell line. Moreover, the antimicrobial activity of all the new analogues was evaluated against a panel of Gram-positive and Gram-negative bacteria, yeast and fungi in comparison to streptomycin and amphotericin-B as reference drugs. Interestingly, both 1 and 3c showed the most promising microbial inhibitory effect. Molecular docking studies showed promising binding patterns of the compounds 1 and 3c with the prospective targets, EGFR, VEGFR-2 and BRAF^V600E^. Finally, additional toxicity studies were performed for the new derivatives which showed their good drug-like properties and low toxicity risks in humans.

## Abstract 2

Deregulation of various protein kinases is considered as one of the important factors resulting in cancer development and metastasis, thus multi-targeting the kinase family is one of the most important strategies in current cancer therapy.

## Graphical abstract

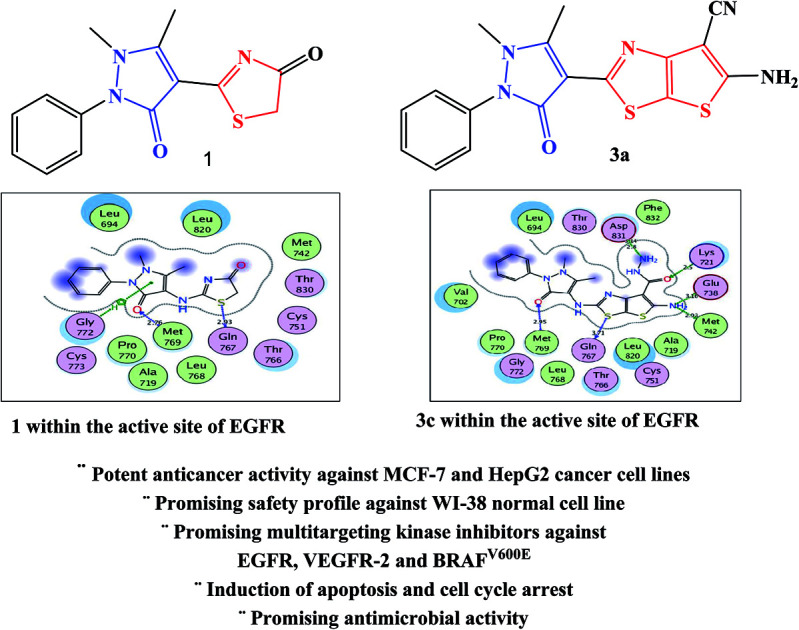

## 1. Introduction

Despite the extensive research and rapid progress in drug science and chemotherapeutic agents for combating cancer, it is still one of the most leading causes of death worldwide.^1^ Statistics show that by 2040, cancer incidence will continue to rise to up to 29.5 million cases per year.^2^ Cancer treatment is still a major issue, owing to the toxicity, resistance, and lack of selectivity of the currently available anticancer medications.^3^ Dysregulated kinase function, which typically acts as an on/off switch for cellular proliferation and motility, is considered as one of the reasons for cancer. Mutation of kinases is responsible for cellular abnormalities leading to cancer initiation, progression or metastasis.^4^ Single and multiple kinase inhibitors are now considered as targeted therapeutic strategies for human malignancy treatment.^5^ Current drug discovery research shows that PIK3CA, BRAF, VEGR and epidermal growth factor receptor (EGFR) are key oncogenic kinase drug targets.^5–8^

Epidermal growth factor receptor (EGFR) is a trans-membrane glycoprotein, belonging to a family which consists of four related receptor tyrosine kinases. It plays a key mediating role in cell signaling pathways including cell proliferation, apoptosis, angiogenesis, and metastatic spread.^9^ This critical role makes it a prominent target in cancer treatment. The overexpression of EGFR in a variety of human cancers, including head, neck, breast, lung, colorectal, prostate, renal, pancreas, ovary, and brain cancers results in poor treatment outcomes due to resistance to hormone therapy, cytotoxic drugs, and radiotherapy.^10,11^ As a result, international recommendations advocate anti-EGFR medicines as the first-line treatment in patients with advanced EGFR mutations, due to their higher efficacy and safety compared to standard chemotherapy.^9,12,13^

Furthermore, vascular endothelial growth factor receptor-2 (VEGFR-2), a transmembrane tyrosine kinase receptor, has been has been identified as the most important factor in inducing angiogenesis,^14–16^ which is considered as one of the defining features of tumor growth, invasion and metastasis. VEGFR-2 has long been recognized as the most important target in cancer anti-angiogenesis therapy.^17,18^ Several small molecule VEGFR-2 inhibitors were clinically approved or evaluated for cancer treatment.^19–21^

RAF is serine/threonine kinases which regulate ERK–MAPK pathway. There are three unique subtypes of RAF kinases in addition to homologues of BRAF.^22–24^ Many studies concluded that the BRAF serine/threonine kinase alterations have been detected in various types of human cancers that are linked to cell growth, survival and differentiation.^25^ BRAF gene (V600E) is the most abundant type of BRAF mutation in human cancers because of substitution of a valine for glutamic acid at position 600. Current researches showed that suppression of BRAF is a new era in human cancer therapeutic treatment.^26^

Drug discovery researches evidenced that heteroaromatic structures resemble various biologically active moieties in human bodies, like nucleic acids, hormones, and neurotransmitters. Accordingly, these scaffolds have been greatly utilized for designing different anticancer compounds which bind with different targets interrupting the biological pathways involved in cancer progress, making them as magnificent starting points for anticancer drug development.^27^

**Fig. 1:**
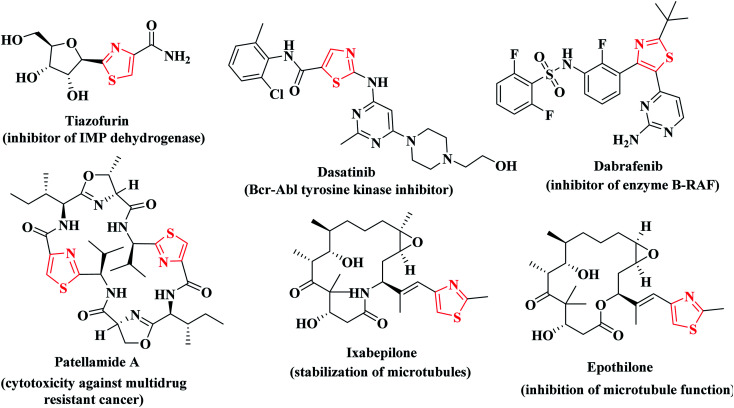
Some clinically approved thiazole-bearing anticancer drugs.

The drug design field ensures that thiazole is the most effective motif for the said target activity. Thiazoles have a superior anticancer activity because they have a better binding domain and less cytotoxicity in normal cells (physiological cells), but they also have site-specific mobility towards the malignant cells (pathological cell).^28^ Additionally, thiazole derivatives were reported to exert cytotoxic potency against several types of cancer disease *via* suppression of various kinases such as; JAK2 and EGFR, VEGFR and BRAF kinases.^29–33^ In addition, different researches confirmed the potent anticancer activities of the compounds containing thiophene and thiazole heterocycles *via* inhibition of BRAF kinase activity.^34,35^ Consequently, the combined substructures (thiophene and thiazole scaffolds) may produce synergistic effects to boost the anticancer activities without compromising their original effective qualities.^27–29^ Many clinically available thiazole-bearing antiproliferative drugs have marked their presence in the field of cancer chemotherapy depicting their anticancer activity profile through diverse mechanisms such as, tiazofurin,^36^ dasatinib,^37^ dabrafenib,^38^ patellamide A,^39^ ixabepilone and epothilone^40,41^ (Fig. 1).

**Fig. 2:**
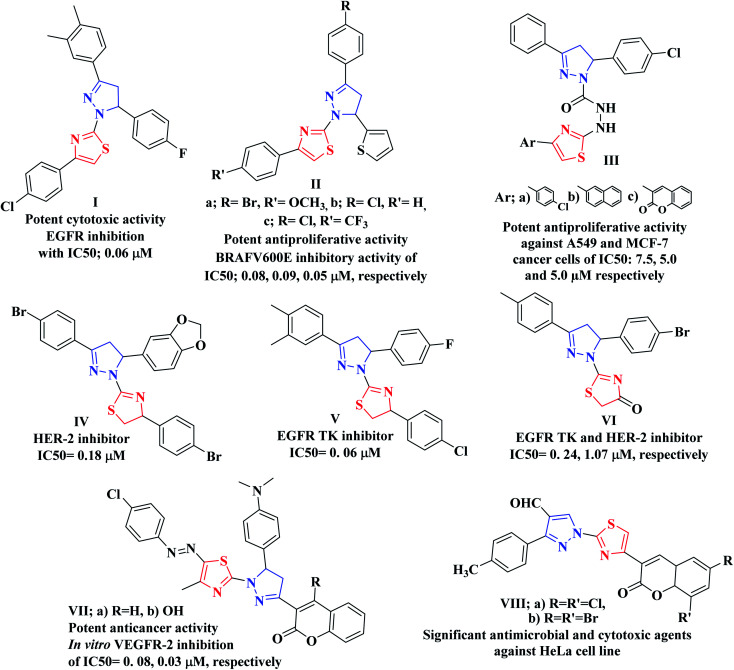
Examples of various thiazolyl-pyrazoline-based compounds as anticancer candidates of TK inhibition activity.

In addition, pyrazole heterocycle is a privileged scaffold possessing diverse biological activities.^42,43^ It is emerged as a useful pharmacophore in the synthesis of potent anticancer drugs.^44,45^ Moreover, multiple studies identified various thiazolyl-pyrazole compounds as potential anticancer agents. As an example, Lv and coworkers have reported that the thiazolyl-pyrazoline derivative I displayed potent cytotoxic activity against breast cancer cell line (MCF-7) with IC_50_ of 0.07 μM *via* EGFR inhibition with IC_50_; 0.06 μM.^46,47^ On the other hand, Zhao *et al.* concluded that the 4,5-dihydropyrazole derivatives bearing thiazole and thiophene moieties IIa–c produced effective antiproliferative activity against WM266.4 and MCF-7 cell lines through BRAF^V600E^ inhibitory activity.^48^ While Sadashiva and coworkers have investigated the dual anticancer activity against A549 and MCF-7 human cancer cell lines as well as antimicrobial activity of the derivatives bearing thiazole heterocycle linked with pyrazoline moiety *via* carbohydrazide linker as compounds IIIa–c.^49^ Furthermore, the thiazoline-pyrazolines IV–VI represented promising cytotoxic effects and different RTKs suppressing impact while, the thiazolopyrazolyl coumarin derivatives VIIa, b exhibited significant *in vitro* anticancer potentiality against different human cancer cell lines *via* a remarkable inhibition of VEGFR-2 with no noticeable toxicity towards the normal cells HFB4.^50^ In addition, Vaarla *et al.* have designed and synthesized the thiazolyl-3-arylpyrazole-4-carbaldehydes VIIIa, b as significant antimicrobial and cytotoxic agents against HeLa cell line and the docking simulation study validated different types of interactions with human microsomal cytochrome P450 (1z11.pdb) enzyme^51^ (Fig. 2).

**Fig. 3:**
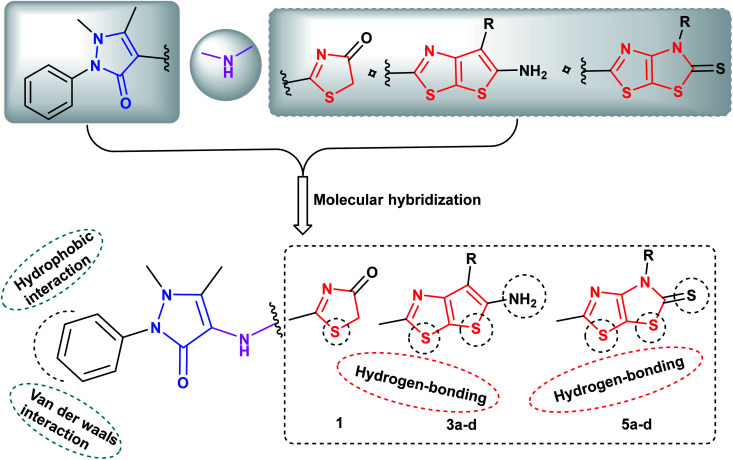
Proposed hypothetic model for pyrazolinyl-thieno[3,2-*d*]thiazole- and pyrazolinyl-thiazolo[4,5-*d*]thiazolidine-2-thione compounds 3a–d and 5a–d, respectively.

Molecular hybridization of two or more bioactive pharmacophores in the same molecular architecture represents an optimistic strategy in discovery of novel anticancer drugs. This approach raises the possibility to synergize the anticancer efficacy *via* targeting two or more molecular proteins as a single entity, reduces the risk of drug–drug interactions as well as prevents the drug resistance obstacle.^52^ Prompted by the above considerations and in view of our continuous efforts in synthesis of heterocyclic derivatives of anticancer activity targeting different protein kinases,^53–56^ the ongoing study was focused on designing and synthesis of two sets of new analogues bearing pyrazoline-3-one ring conjugated either with the fused thieno[3,2-*d*]thiazole scaffold 3a–c or with dihydrothiazolo[4,5-*d*]thiazole scaffold 5a–c*via* NH linker as potential anticancer agents of multi-targeted tyrosine kinase inhibiting activity against EGFR, VEGFR-2 and BRAF^V600E^ kinases. It was taken into account the impact of molecular orientation, ring size variation and the presence of heteroatoms that could participate in hydrogen bonding interactions with the binding pockets of the protein kinases under study (Fig. 3).

All the newly synthesized compounds were assessed as anticancer candidates against human breast cancer cells (MCF-7) and human liver carcinoma cell line (HepG-2). The safety of the most promising anticancer candidates was also evaluated against the normal WI-38 cell line. Moreover, the most promising cytotoxic compounds were further evaluated as multitargeting protein kinases against EGFR, VEGFR-2 and f BRAF^V600E^ and were investigated for their impact on cell cycle and apoptosis induction potential in MCF-7 cell line.

Immunosuppression which usually results due to anticancer drug regimen and destruction of the mucosal barrier due to utilizing invasive devices in cancer patients make them more vulnerable to different infectious diseases that need long-term prophylactic antibiotic regimens.^57^ Moreover, the efficiency of antibiotic regimen to combat infections in cancer patients is compromised due to the growth of drug resistant bacterial pathogens that dominate in neutropenic patients.^58^ Accordingly, novel anticancer agents that specifically suppress cancer cells having dual anticancer and antimicrobial activities are considered to act as prophylaxis against microbial infections alongside suppressing the growth of tumors in cancer patients.^57,58^ Accordingly, the new compounds were further subjected to antimicrobial investigation in comparison to amoxicillin trihydrate and clotrimazole as standard antibacterial and antifungal drugs, respectively, against a panel of Gram-positive, Gram-negative bacterial, yeast and fungal strains. In addition, minimum inhibitory concentrations were also assessed for the new candidates. Additional toxicity study was performed for the new derivatives which represented their good drug-likeness properties and low toxicity risks in humans.

## 2. Experimental

### 2.1. Chemistry

The instruments used for measuring the melting points, spectral data (IR, mass, ^1^H NMR and ^13^C NMR) and elemental analyses are provided in details in ESI.†

The chemical names given for the prepared compounds are according to the IUPAC system. 2-Chloro-*N*-(1,5-dimethyl-3-oxo-2-phenyl-2,3-dihydro-1*H*-pyrazol-4-yl)acetamide was prepared according to the reported method.^59^

#### 2.1.1. 2-((1,5-Dimethyl-3-oxo-2-phenyl-2,3-dihydro-1*H*-pyrazol-4-yl)amino)thiazol-4(5*H*)-one (1)

A mixture of 2-chloro-*N*-(1,5-dimethyl-3-oxo-2-phenyl-2,3-dihydro-1*H*-pyrazol-4-yl)acetamide (10 mmol) and ammonium thiocyanate (10 mmol) in absolute ethanol (20 mL) was refluxed for 4 h and the obtained precipitate during reflux was filtered, washed with water, dried and recrystallized from ethanol to give the title compound 1 as white crystals.

Yield 86%, mp 242–244 °C; IR (*ν*_max_/cm^−1^): 3382 (NH), 3050 (CH-arom.), 2945 (CH-aliph.), 1685, 1655 (2CO); ^1^H NMR (DMSO-*d*_6_) *δ*: 2.15 (s, 3H, CH_3_), 3.21 (s, 3H, NCH_3_), 4.06 (s, 2H, CH_2_), 7.05–7.53 (m, 5H, Ar-H), 9.62 (s, 1H, NH, D_2_O exchangeable); ^13^C NMR (DMSO-*d*_6_): 12.11 (CH_3_), 32.04 (CH_3_), 35.16 (CH_2_), 116.10, 121.07, 122.69, 128.59, 131.84, 137.91, 158.00, 166.74 (C=O), 176.13 (C=O); MS, *m*/*z* (%): 302 [M^+^] (14), 77 (100%); anal. calcd for C_14_H_14_N_4_O_2_S (302.35): C, 55.61; H, 4.67; N, 18.53; S, 10.61%. Found: C, 55.83; H, 4.89; N, 18.75; S, 10.84%.

#### 2.1.2. General procedure for the synthesis of thieno[3,2-*d*]thiazole derivatives 3a–d

To a mixture of thiazolidin-4-one derivative 1 (10 mmol, 3.02 g) in absolute ethanol (25 mL) and dimethylformamide (2 mL) containing triethylamine (1 mL), active methylene compound 2a–d namely; malononitrile, ethyl cyanoacetate, cyanoacetohydrazide and/or cyanothioacetamide (10 mmol) was added followed by the addition of an equimolar amount of elemental sulfur (10 mmol, 0.32 g). The reaction mixture was heated under reflux for 7 h, then cooled and neutralized by pouring onto ice/cold water containing few drops of hydrochloric acid. The formed precipitate was filtered, dried and recrystallized from ethanol.

##### 2.1.2.1. 5-Amino-2-((1,5-dimethyl-3-oxo-2-phenyl-2,3-dihydro-1*H*-pyrazol-4-yl)amino)thieno [3,2-*d*]thiazole-6-carbonitrile (3a)

Yield: 72%; brown crystal; mp >300 °C; IR (*ν*_max_/cm^−1^): 3415, 3373 (NH_2_), 3226 (NH), 3067 (CH-arom.), 2985 (CH-aliph.), 2212 (CN), 1658 (CO); ^1^H NMR (DMSO-*d*_6_) *δ*: 2.15 (s, 3H, CH_3_), 3.07 (s, 3H, NCH_3_), 6.44 (s, 2H, NH_2_, D_2_O exchangeable), 7.35–7.58 (m, 5H, Ar-H), 9.25 (s, 1H, NH, D_2_O exchangeable); ^13^C NMR (DMSO-*d*_6_): 15.62 (CH_3_), 33.83 (CH_3_), 106.91, 109.67, 115.86, 118.20, 120.01, 122.69, 129.60, 130.74, 135.94, 139.17, 153.27, 156.13, 165.83 (C=O); MS, *m*/*z* (%): 382 [M^+^] (44), 77 (100); anal. calcd for C_17_H_14_N_6_OS_2_ (382.46): C, 53.39; H, 3.69; N, 21.97; S, 16.77%. Found: C, 53.60; H, 3.89; N, 21.74; S, 16.56%.

##### 2.1.2.2. Ethyl 5-amino-2-((1,5-dimethyl-3-oxo-2-phenyl-2,3-dihydro-1*H*-pyrazol-4-yl)amino) thieno[3,2-*d*]thiazole-6-carboxylate (3b)

Yield: 75%; pale brown crystal; mp 285–287 °C; IR (*ν*_max_/cm^−1^): 3392, 3318 (NH_2_), 3237 (NH), 3057 (CH-arom.), 2942 (CH-aliph.), 1715, 1654 (2CO); ^1^H NMR (DMSO-*d*_6_) *δ*: 1.23 (t, *J* = 7.2 Hz, 3H, OCH_2_
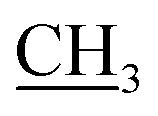
), 2.18 (s, 3H, CH_3_), 3.11 (s, 3H, NCH_3_), 4.15 (q, *J* = 7.2 Hz, 2H, CH_2_, O
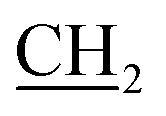
CH_3_), 6.70 (s, 2H, NH_2_, D_2_O exchangeable), 7.39–7.61 (m, 5H, Ar-H), 9.27 (s, 1H, NH, D_2_O exchangeable); ^13^C NMR (DMSO-*d*_6_): 12.35 (OCH_2_
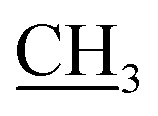
), 16.24 (CH_3_), 31.70 (NCH_3_), 62.99 (O
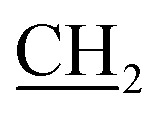
CH_3_), 109.10, 117.58, 120.61, 123.28, 125.01, 129.42, 131.03, 134.19, 139.81, 154.38, 158.18, 165.57 (C=O), 168.73(C=O, ester); MS, *m*/*z* (%): 429 [M^+^] (75), 228 (100); anal. calcd for C_19_H_19_N_5_O_3_S_2_ (429.52): C, 53.13; H, 4.46; N, 16.31; S, 14.93%. Found: C, 53.35; H, 4.67; N, 16.54; S, 14.72%.

##### 2.1.2.3. 5-Amino-2-((1,5-dimethyl-3-oxo-2-phenyl-2,3-dihydro-1*H*-pyrazol-4-yl)amino) thieno[3,2-*d*]thiazole-6-carbohydrazide (3c)

Yield: 70%; buff crystal; mp 293–295 °C; IR (*ν*_max_/cm^−1^): 3431, 3356, 3284, 3222 (2NH_2_), 3178 (2NH), 3069 (CH-arom.), 2937 (CH-aliph.), 1698, 1652 (2CO); ^1^H NMR (DMSO-*d*_6_) *δ*: 2.21 (s, 3H, CH_3_), 3.10 (s, 3H, NCH_3_), 5.91 (s, 2H, NH_2_, D_2_O exchangeable), 7.04–7.66 (m, 8H, Ar-H, NH, NH_2_, D_2_O exchangeable), 9.81 (s, 1H, NH, D_2_O exchangeable); ^13^C NMR (DMSO-*d*_6_): 13.75 (CH_3_), 33.92 (NCH_3_), 109.35, 114.51, 119.71, 123.61, 129.95, 131.08, 132.52, 137.40, 139.92, 151.73, 156.24, 166.78 (C=O), 169.89 (C=O); MS, *m*/*z* (%): 415 [M^+^] (15), 214 (100); anal. calcd for C_17_H_17_N_7_O_2_S_2_ (415.49): C, 49.14; H, 4.12; N, 23.60; S, 15.43%. Found: C, 49.35; H, 4.33; N, 23.81; S, 15.64%.

##### 2.1.2.4. 5-Amino-2-((1,5-dimethyl-3-oxo-2-phenyl-2,3-dihydro-1*H*-pyrazol-4-yl)amino) thieno[3,2-*d*]thiazole-6-carbothioamide (3d)

Yield: 73%; brown crystal; mp 297–299 °C; IR (*ν*_max_/cm^−1^): 3423, 3381, 3276, 3215 (2NH_2_), 3155 (NH), 3070 (CH-arom.), 2943 (CH-aliph.), 1661 (CO); ^1^H NMR (DMSO-*d*_6_) *δ*: 2.09 (s, 3H, CH_3_), 3.16 (s, 3H, NCH_3_), 6.82 (s, 2H, NH_2_, D_2_O exchangeable), 7.53–7.99 (m, 7H, Ar-H, NH_2_), 9.78 (s, 1H, NH, D_2_O exchangeable); ^13^C NMR (DMSO-*d*_6_): 12.42 (CH_3_), 32.37 (NCH_3_), 111.66, 117.31, 119.25, 121.95, 123.45, 129.61, 130.11, 132.93, 137.12, 151.32, 154.20, 165.52 (C=O), 183.72 (C=S); MS, *m*/*z* (%): 417 [M^+1^] (29), 56 (100); anal. calcd for C_17_H_16_N_6_OS_3_ (416.54): C, 49.02; H, 3.87; N, 20.18; S, 23.09%. Found: C, 49.25; H, 3.65; N, 20.39; S, 23.31%.

#### 2.1.3. General procedure for synthesis of dihydrothiazolo[4,5-*d*]thiazoles 5a–d

A mixture of thiazolidin-4-one derivative 1 (10 mmol, 3.02 g), isothiocyanate derivatives 4a–d namely; phenyl isothiocyanate, ethyl isothiocyanate, methyl isothiocyanate and/or *p*-chloro phenyl isothiocyanate (10 mmol) and elemental sulfur (10 mmol, 0.32 g) in absolute ethanol (25 mL) and dimethylformamide (2 mL) containing triethylamine (1.0 mL) was heated under reflux for 8 h. After cooling, the reaction mixture was pouring onto ice/cold water and acidified by hydrochloric acid. The formed precipitate was filtered, dried and recrystallized from dioxane.

##### 2.1.3.1. 1,5-Dimethyl-2-phenyl-4-((4-phenyl-5-thioxo-4,5-dihydrothiazolo[4,5-*d*]thiazol-2-yl) amino)-1*H*-pyrazol-3(2*H*)-one (5a)

Yield: 68%; dark brown crystal; mp 201–203 °C; IR (*ν*_max_/cm^−1^): 3335 (NH), 3078 (CH-arom.), 2936 (CH-aliph.), 1657 (CO), 1273 (C=S); ^1^H NMR (DMSO-*d*_6_) *δ* = 2.19 (s, 3H, CH_3_), 3.03 (s, 3H, NCH_3_), 7.05–7.62 (m, 6H, Ar-H), 7.85 (d, *J* = 7.2 Hz, 2H, Ar-H), 7.99 (d, *J* = 7.2 Hz, 2H, Ar-H), 10.13 (s, 1H, NH, D_2_O exchangeable); ^13^C NMR (DMSO-*d*_6_): 13.71 (CH_3_), 34.37 (NCH_3_), 116.48, 119.30, 120.55, 125.12, 127.26, 128.45, 129.76, 132.38, 136.23, 139.48, 143.16, 152.59, 157.38, 164.50 (C=O), 185.12 (C=S); MS, *m*/*z* (%): 451 [M^+^] (30), 452 [M^+1^] (25), 249 (100); anal. calcd for C_21_H_17_N_5_OS_3_ (451.59): C, 55.85; H, 3.79; N, 15.51; S, 21.30%. Found: C, 55.64; H, 3.57; N, 15.73; S, 21.51%.

##### 2.1.3.2. 4-((4-Ethyl-5-thioxo-4,5-dihydrothiazolo[4,5-*d*]thiazol-2-yl)amino)-1,5-dimethyl-2-phenyl-1*H*-pyrazol-3(2*H*)-one (5b)

Yield: 65%; brown crystal; mp 183–185 °C; IR (*ν*_max_/cm^−1^): 3352 (NH), 3085 (CH-arom.), 2978 (CH-aliph.), 1656 (CO), 1247 (C=S); ^1^H NMR (DMSO-*d*_6_) *δ*: 1.22 (t, *J* = 7.2 Hz, 3H, –CH_2_
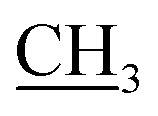
), 2.65 (s, 3H, CH_3_), 3.01 (s, 3H, NCH_3_), 4.15 (q, *J* = 7.2 Hz, 2H, 
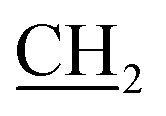
–CH_3_), 7.41–7.98 (m, 5H, Ar-H), 10.05 (s, 1H, NH, D_2_O exchangeable); ^13^C NMR (DMSO-*d*_6_): 13.92 (–CH_2_
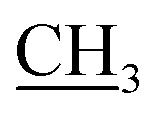
), 18.35 (CH_3_), 32.66 (NCH_3_), 51.20 (
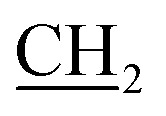
CH_3_), 118.19, 121.64, 124.03, 127.46, 130.63, 135.26, 146.10, 157.71, 157.96, 164.56 (C=O), 184.27 (C=S); MS, *m*/*z* (%): 404 [M^+1^] (28), 327 (100); anal. calcd for C_17_H_17_N_5_OS_3_ (403.54): C, 50.60; H, 4.25; N, 17.35; S, 23.84%. Found: C, 50.82; H, 4.46; N, 17.57; S, 23.63%.

##### 2.1.3.3. 1,5-Dimethyl-4-((4-methyl-5-thioxo-4,5-dihydrothiazolo[4,5-*d*]thiazol-2-yl)amino)-2-phenyl-1*H*-pyrazol-3(2*H*)-one (5c)

Yield: 61%; reddish brown crystal; mp 189–191 °C; IR (*ν*_max_/cm^−1^): 3328 (NH), 3074 (CH-arom.), 2983 (CH-aliph.), 1652 (CO), 1261 (C=S); ^1^H NMR (DMSO-*d*_6_) *δ*: 2.27 (s, 3H, CH_3_), 3.05 (s, 3H, NCH_3_), 3.06 (s, 3H, NCH_3_), 7.33–7.68 (m, 5H, Ar-H), 10.14 (s, 1H, NH, D_2_O exchangeable); ^13^C NMR (DMSO-*d*_6_): 11.89 (CH_3_), 31.74 (NCH_3_), 34.38 (NCH_3_), 117.01, 121.82, 123.94, 128.43, 131.24, 138.42, 143.51, 150.29, 158.32, 165.06 (C=O), 187.76 (C=S); MS, *m*/*z* (%): 389 [M^+^] (55), 188 (100); anal. calcd for C_16_H_15_N_5_OS_3_ (389.52): C, 49.34; H, 3.88; N, 17.35; S, 24.70%. Found: C, 49.56; H, 3.67; N, 17.76; S, 24.91%.

##### 2.1.3.4. 4-((4-(4-Chlorophenyl)-5-thioxo-4,5-dihydrothiazolo[4,5-*d*]thiazol-2-yl)amino)-1,5-dimethyl-2-phenyl-1*H*-pyrazol-3(2*H*)-one (5d)

Yield: 69%; brown crystal; mp 211–213 °C; IR (*ν*_max_/cm^−1^): 3320 (NH), 3089 (CH-arom.), 2943 (CH-aliph.), 1662 (CO), 1287 (C=S); ^1^H NMR (DMSO-*d*_6_) *δ*: 2.12 (s, 3H, CH_3_), 3.02 (s, 3H, NCH_3_), 6.98 (d, *J* = 12.9 Hz, 2H, Ar-H), 7.29–7.34 (m, 1H, Ar-H), 7.52–7.78 (m, 6H, Ar-H), 9.43 (s, 1H, NH, D_2_O exchangeable); ^13^C NMR (DMSO-*d*_6_): 12.18 (CH_3_), 32.86 (NCH_3_), 119.81, 122.42, 125.11, 128.02, 129.22, 130.16, 131.06, 133.82, 138.45, 141.73, 145.51, 158.18, 161.92, 165.87 (C=O), 183.79 (C=S); MS, *m*/*z* (%): 487, 485 [M^+^] (25, 70), 202 (100); anal. calcd for C_21_H_16_ClN_5_OS_3_ (486.03): C, 51.89; H, 3.32; Cl, 7.29; N, 14.41; S, 19.79%. Found: C, 51.68; H, 3.55; Cl, 7.51; N, 14.63; S, 19.58%.

### 2.2. Biological activity

#### 2.2.1. *In vitro* anticancer screening

The cell lines were purchased from the American Type Culture collection as follows: liver carcinoma cell line (HepG-2) and breast carcinoma cell line (MCF-7). Cytotoxic activity screening was performed using MTT assay at Regional Center for Mycology and Biotechnology, Al-Azhar University.^60^ More details in the ESI.†

#### 2.2.2. EGFR, VEGFR and BRAF^V600E^ kinase inhibitory assay

The most active cytotoxic compounds that showed promising IC_50_ values against 1, 3c were further examined for their inhibitory activities against EGFR, VEGFR-2 and BRAF^V600E^.^61–63^ More details in the ESI.†

#### 2.2.3. *In vitro* DNA-flow cytometric (cell cycle) analysis

To determine the distribution of cell lines in each phase of cell cycle, the PI was used to stain the DNA content of each cell line. At a density of 1 × 10^6^ to 3 × 10^6^ cells per dish, MCF-7 cells were seeded in 30 mm tissue culture plates in 5 mL of complete medium.^63^ More details in the ESI.†

#### 2.2.4. Annexin V-FITC apoptosis assay

Annexin V-FITC apoptosis detection kit (BD biosciences) was used to quantify the percentage of cells undergoing apoptosis and to determine the mode of cell death whether by apoptosis or necrosis in the presence or absence of the active compounds 1, 3c. The experiment was carried out according to the manufacturer's protocol.^63^ More details in the ESI.†

#### 2.2.5. Antimicrobial activity assay

*In vitro* microbial activities were carried out at the Regional Center for Mycology and Biotechnology (RCMB), Al-Azhar University, Cairo, Egypt. The biological potential of the newly prepared target structures was inspected toward the examined organisms and expressed as the diameter of the inhibition zones due to the agar plate diffusion technique.^64–69^ More details in the ESI.†

##### 2.2.5.1. Minimal inhibitory concentration (MIC) measurement

The bacteriostatic activity of the compounds was then evaluated using the two-fold serial dilution technique. Two-fold serial dilutions of the tested compounds solutions were prepared using the proper nutrient broth.^67–69^ More details in the ESI.†

### 2.3. Computational studies

#### 2.3.1. Molecular docking

The 2D structure of the newly synthesized derivatives 1 and 3c was drawn through ChemDraw. The protonated 3D was employed using standard bond lengths and angles, using Molecular Operating Environment (MOE-Dock) software version 2014.0901.^70–72^ Then, the geometry optimization and energy minimization were applied to get the Conf Search module in MOE, followed by saving of the MOE file for upcoming docking process. The co-crystallized structures of EGFR, VEGFR-2 and BRAF^V600E^ kinases with their ligands erlotinib, sorafenib and SB-590885 were downloaded (PDB codes: 1M17, 4ASD and 2FB8, respectively) from protein data bank.^73–75^ More details in the ESI.†

#### 2.3.2. *In silico* toxicity potential

Toxicity risks and physicochemical characteristics were given for the newly synthesized derivatives following Osiris methodology and mentioned in details within the ESI.†^76^

## 3. Results and discussion

### 3.1. Chemistry

**Scheme 1:**
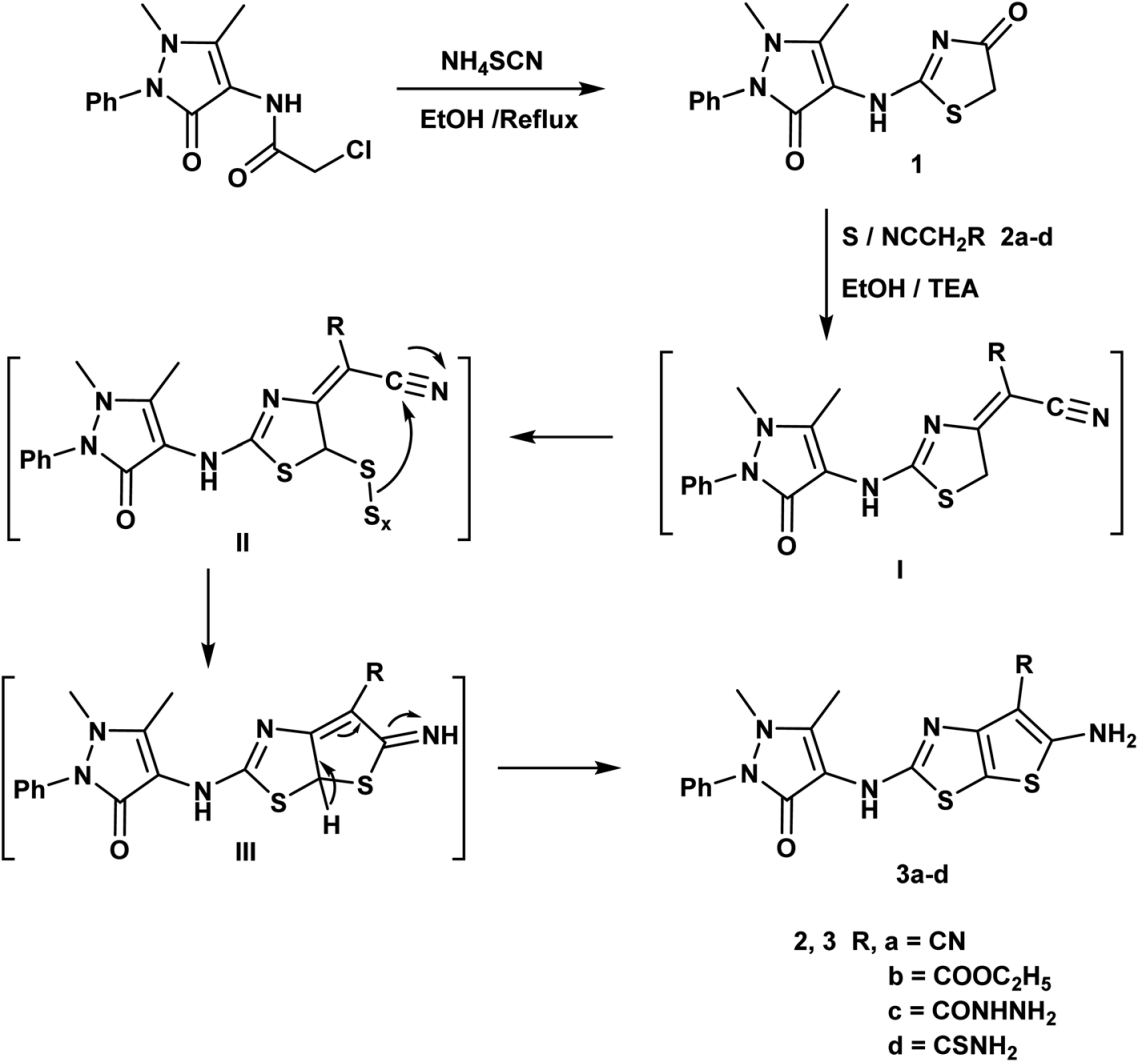
Synthesis of thienothiazole derivatives.

**Scheme 2:**
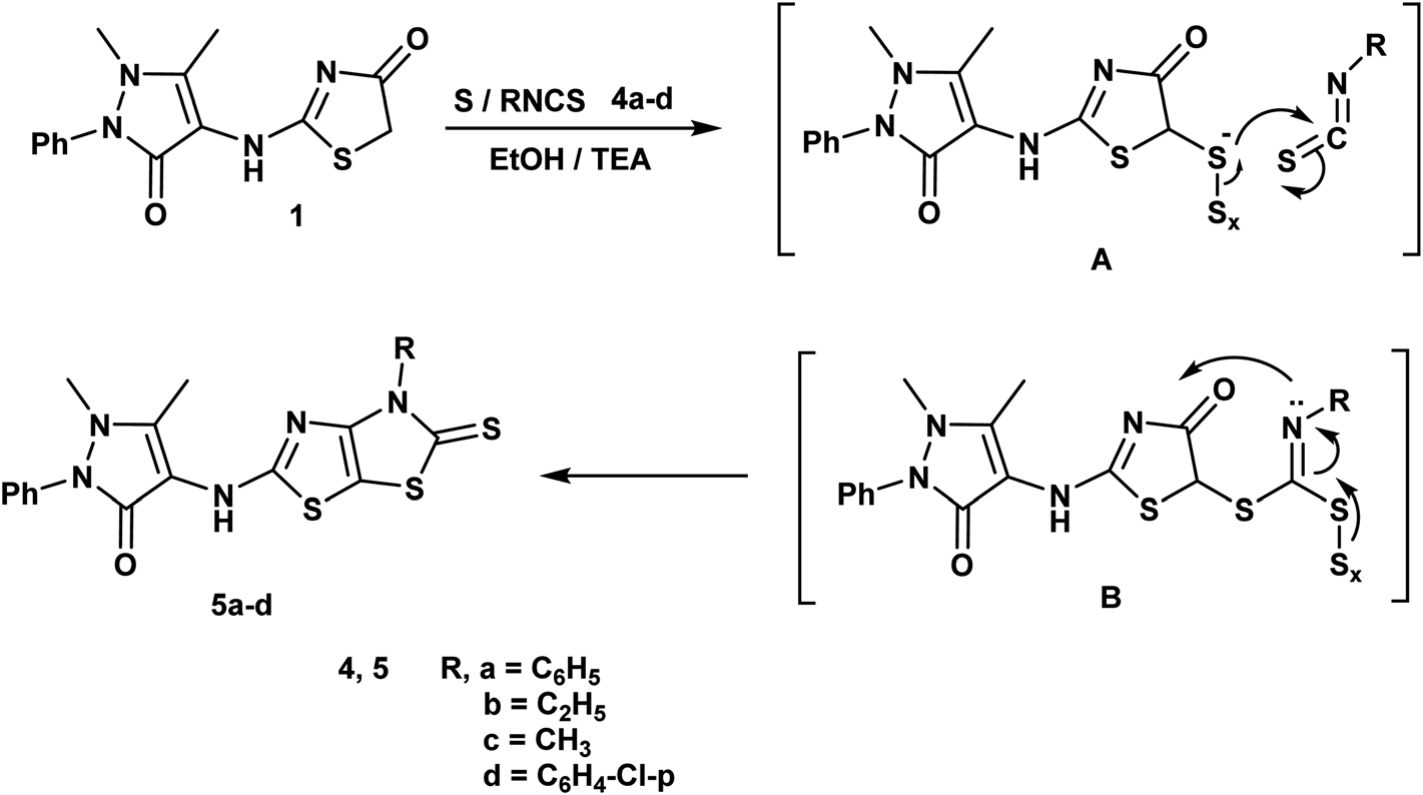
Synthesis of thiazolothiazole derivatives.

An efficient synthesis of the new thienothiazoles 3a–d and thiazolothiazoles 5a–d has been performed starting with (1,5-dimethyl-3-oxo-2-phenyl-2,3-dihydro-1*H*-pyrazol-4-yl)carbamic chloride.^59^ Their synthetic routes were outlined in (Schemes 1 and 2). Refluxing 2-chloro-*N*-(1,5-dimethyl-3-oxo-2-phenyl-2,3-dihydro-1*H*-pyrazol-4-yl)acetamide with ammonium thiocyanate in ethanol produced the corresponding thiazolidin-4-one derivative 1. The ^1^H-NMR spectrum of compound 1 appeared three singlet signals at *δ* 2.15, 3.21 and 4.06 ppm due to CH_3_, NCH_3_ and CH_2_ respectively and also, its ^13^C-NMR spectrum showed signals at *δ* 12.11, 32.04 and 35.16 ppm due to CH_3_, NCH_3_ and CH_2_ respectively.

The Gewald reaction is a condensation of ketone or aldehyde with acetonitriles substituted by a strong electron withdrawing group (such as ester, amide, and nitrile) in the presence of elemental sulfur and base to give a poly-substituted 2-amino-thiophene. So, the thieno[3,2-*d*]thiazole derivatives 3a–d were obtained *via* the treatment of key compound 1 according to Gewald reaction with a series of active methylene derivatives namely; malononitrile, ethyl cyanoacetate, cyanoacetohydrazide and/or cyanothioacetamide in the presence of sulfur and triethylamine. IR spectrum of the compound 3a showed bands at 3415, 3373 cm^−1^ referring to NH_2_ and strong absorption band at 2212 cm^−1^ due to CN. The ^1^H-NMR spectrum of compound 3b displayed triplet and quartet signals at *δ* 1.23 and 4.15 ppm according to the ester group (Scheme 1).

Furthermore, reaction of thiazolidin-4-one moiety 1 with different isothiocynate derivatives namely; phenyl isothiocyanate, ethyl isothiocyanate, methyl isothiocyanate and/or *p*-chloro phenyl isothiocyanate in the presence of sulfur and triethylamine afforded the corresponding dihydrothiazolo[4,5-*d*]thiazole derivatives 5a–d respectively. The ^1^H-NMR spectrum of compound 5b displayed triplet and quartet signals at *δ* 1.22 and 4.15 ppm according to the ethyl group and its 13C-NMR showed signals at *δ* 13.92, 51.20 and 184.27 ppm referring to ethyl and C=S groups (Scheme 2).

### 3.2. Biological activity

#### 3.2.1. *In vitro* anti-proliferative activity

The cytotoxic activity of the newly synthesized compounds was assessed against two cancer cell lines; human breast adenocarcinoma (MCF-7) and hepatocellular carcinoma (HepG-2) using MTT assay.^60^ These types of cancer cell lines have a high expression of EGFR, VEGFR and BRAF.^10,11,17,18,26^ In addition, the cytotoxic effect of the new compounds was also evaluated against the normal lung fibroblast (WI38) to demonstrate their safety profiles. The commercially available doxorubicin was utilized as a standard drug. The resultant data represented the concentrations of the derivatives that led to 50% inhibition of cell viability (IC_50_, μM) which were reported in Table 1. According to IC_50_ values in Table 1 it has been revealed that the evaluated compounds exhibited versatile anti-proliferative activities against the cell lines under study. Interestingly, the starting pyrazolinyl-aminothiazol-4-one 1 produced the most significant cytotoxic activity. It evidently reduced the viability of both MCF-7 and HepG-2 in an equipotent manner to that of doxorubicin with IC_50_ values of 4.02 ± 0.02, 4.52 ± 0.04 μM, respectively, IC_50 doxorubicin_; 4.62 ± 0.0, 5.66 ± 0.01 μM, respectively. A mild decrease in the activity against both cancer cell lines (about 1.8 and 1.3 folds, respectively) was detected by the thieno[3,2-*d*]thiazole-carbohydrazide derivative 3c of IC_50_ values; 8.35 ± 0.03 and 7.88 ± 0.01 μM, respectively. The carbohydrazide side chain produced an important point for hydrogen binding interaction with the target proteins. On the other hand, the rest of compounds showed moderate to weak cytotoxic activities. This result indicated that the conjugation of the thiazolinone moiety with the pyrazolinone nucleus produced a significant cytotoxic potency, while the fused thieno[3,2-*d*]thiazole or dihydrothiazolo[4,5-*d*]thiazole scaffolds negatively affect the activity, which might be due to improper fitting in the active sites of the proteins which they act on.

**Table 1:** *In vitro* cytotoxic efficiency of the new derivatives against MCF-7 as well as HepG-2 cell lines

Compound no.	IC_50_^a^ (μM)	IC_50_^a^ (μM)	IC_50_^a^ (μM)
	MCF-7	HepG-2	WI-38
1	4.02 ± 0.02	4.52 ± 0.04	60.44
3a	27.58 ± 0.01	25.72 ± 0.03	
3b	36.77 ± 0.02	39.02 ± 0.02	
3c	8.35 ± 0.03	7.88 ± 0.01	54.72
3d	18.19 ± 0.01	16.91 ± 0.01	
5a	73.80 ± 0.04	65.19 ± 0.02	
5b	43.94 ± 0.02	48.04 ± 0.03	
5c	14.69 ± 0.03	11.85 ± 0.03	
5d	88.56 ± 0.01	81.50 ± 0.03	
Doxorubicin	4.62 ± 0.01	5.66 ± 0.01	

a IC_50_ values = mean ± SD of three independent determinations.

The safety of the most active compounds 1, 3c towards the normal fibroblasts (WI-38) cell line was also examined. The tested compounds were found to be more selective to MCF-7 and HepG-2 cancer cell lines than to the normal cell line WI-38 as they revealed cytotoxicity with IC_50_ of 60.44, 54.72 μM. Therefore, they can be considered to be safe candidates against the normal cell line.

#### 3.2.2. The inhibitory effect of compounds 1, 3c towards EGFR, VEGFR-2 and BRAF^V600E^ tyrosine kinases

The most active cytotoxic agents 1, 3c were also evaluated as multitargeted protein kinases against EGFR, VEGFR-2 and BRAF^V600E^ tyrosine kinases and sorafenib was used as a positive control.^61,62^ The obtained results were summarized in Table 2. A significant sensitivity was detected by EGFR kinase against both compounds 1, 3c which revealed a near equipotent or slightly higher inhibitory activity than that of the reference drug sorafenib with IC_50 s_; 0.022, 0.017 μM, respectively, while IC_50 sorafenib_; 0.025 μM. On the other hand, about 2.4–2.2 folds decrease in the inhibitory activity was detected by the tested derivatives 1, 3c against the target VEGFR-2 while of IC_50 s_; 2.470, 2.259 μM, respectively comparing to sorafenib with IC_50_; 1.022 μM. Furthermore, the thieno[3,2-*d*]thiazole derivative 3c exhibited a promising inhibitory activity against BRAF^V600E^ but slightly less than that of standard drug sorafenib with IC_50 s_; 0.088, 0.040 μM, respectively. On the other hand, a remarkable drop in the activity was detected by the pyrazolinyl-thiazolinone derivative 1 against BRAF^V600E^ employing IC_50_; 2.026 μM. It could be noticed that the bicyclic thieno-thiazole pharmacophore produced a positive impact towards the inhibition effect against target BRAF^V600E^ kinase.

**Table 2:** Inhibitory assessment of the tested compounds 1 and 3c in comparison with sorafenib against EGFR, VEGFR-2 and BRAF^V600E^^a^

Compound no.	IC_50_ (mean ± SEM) (μM)		
	EGFR	VEGFR-2	BRAF^V600E^
Sorafenib	0.025 ± 0.10	1.022 ± 0.15	0.040 ± 0.13
1	0.022 ± 1.00	2.470 ± 0.30	2.026 ± 0.50
3c	0.017 ± 0.05	2.259 ± 0.45	0.088 ± 0.01

a IC_50_: compound concentration required to inhibit the enzyme activity by 50%, SEM: standard error mean; each value is the mean of three values.

#### 3.2.3. *In vitro* DNA-flow cytometric (cell cycle) analysis

**Fig. 4:**
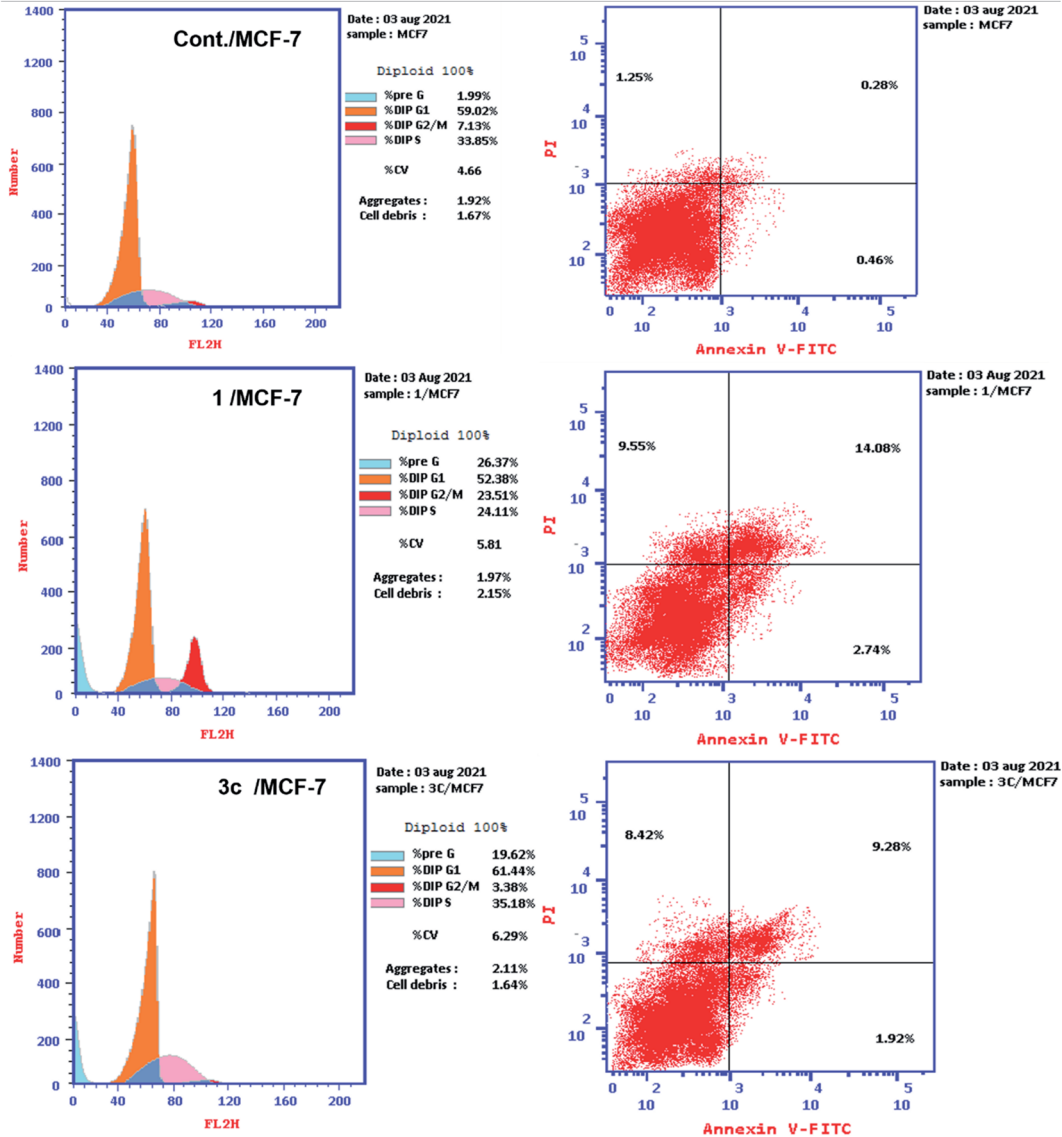
Cell cycle analysis of and activation of apoptosis caused by the derivatives 1 and 3c against MCF-7 cells.

The most potent analogues 1, 3c were selected to study their effects on cell cycle progression in MCF-7 cell line. DMSO was utilized as a negative control. The stages of cell cycle were being detected *via* flow cytometry after propidium iodide (PI) staining followed by RNAse treatment.^63^ In this experiment, MCF-7 cells were incubated with a concentration of 4.021 μM for compound 1 and with 8.35 μM for compound 3c for 24 h. The flow cytometry approach was used to quantify cell populations at various stages of the cell cycle (pre-G1, G1, S and G2/M phases). The obtained results indicated that MCF-7 cells treatment with compounds 1 and 3c led to an interference with the normal cell cycle distribution. The compound 1 produced a remarkable increase in the percentages of the cells at pre-G1 and G2/M phases from 1.99% and 7.31%, respectively in the control cells to reach to 26.37% and 23.51% in MCF-7 cells treated with compound 1. Such increase was accompanied by an observable reduction in the percentages of cells at G1 and S-stages of the cell cycle from 59.02% and 33.85% in the control cells to 52.38% and 24.11% in the treated cells. This result clearly investigated that compound 1 arrested the cell cycle at G2/M phase. On the other hand, compound 3c induced an increase in the percentages of cells at pre-G1 and S-phases to reach to 19.62% and 35.18% comparing to the control cells and decreased cell percentage at G2/M phase from 33.85% in control cells to 3.38% in the treated cells. This result represented that there was cell cycle arrest at S phase and cessation of mitotic cycle (Fig. 4).

#### 3.2.4. Determination of apoptosis using annexin V-FITC staining technique

**Fig. 5:**
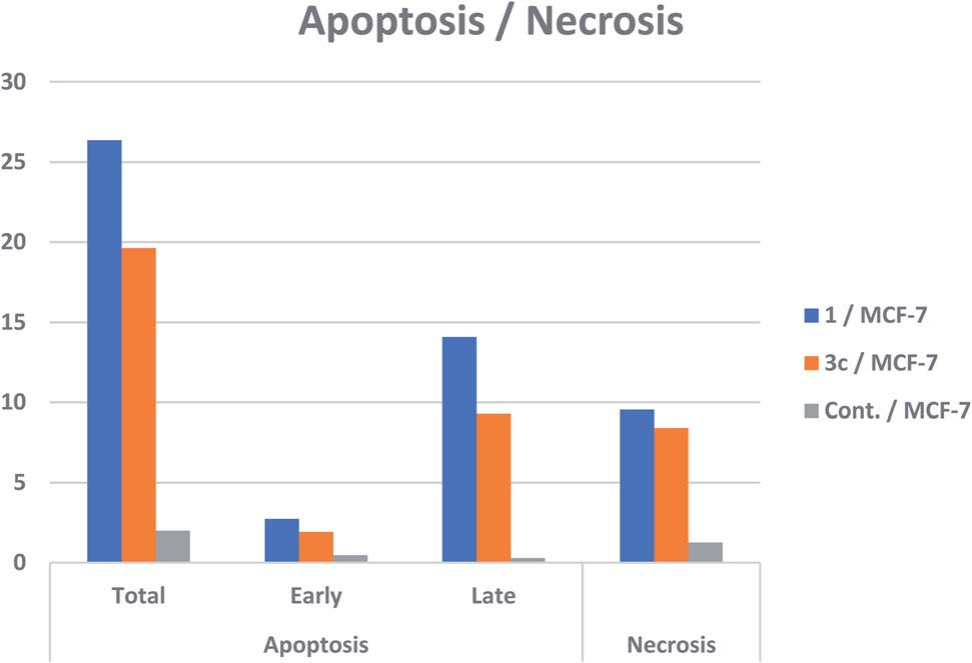
Apoptosis induction induced by the derivatives 1, 3c.

The apoptotic mode of cell death induced by compounds 1 and 3c was further determined to find out whether the cell death was due to apoptosis or necrosis. This assay was carried out using annexin V/PI assay. Annexin V conjugated with FITC was utilized to stain cells in combination with PI.^63^ The cells which stained positive for annexin V/PI indicated the cells in the late apoptotic stage which have lost the integrity of their membranes.^63^ MCF-7 cells were treated with the tested derivatives 1 and 3c at their IC_50_ concentrations 4.021 μM and 8.35 μM, respectively for a period of 24 h. Then staining of MCF-7 cells was carried out using two dyes; annexin V/propidium iodide (PI). Flow cytometry technique has detected the corresponding red (PI) and green (FITC) fluorescence. The representative dot plots of flow cytometric analyses exhibited four various distributions (Fig. 4): lower left (annexin V and PI negative) represents the healthy cells, lower right (annexin V positive and PI negative) represents the cells in early apoptosis, upper right (annexin V and PI positive) represents cells in late apoptosis and finally upper left (annexin V negative and PI positive) dead or necrotic cells. Both compounds have increased the early apoptosis from 0.47% (DMSO control) to 2.74% and 1.92%, respectively. Also, there was an increase in the late apoptosis by both 1 and 3c from 0.28% (DMSO control) to 14.08% and 9.28%, respectively with necrotic percent of 9.55% and 8.42% *vs.* 0.66% produced by DMSO control. It was observable that the late apoptosis percentage caused by compounds 1, 3c was higher than that of the early phase making it more challenging to recover the apoptotic cells to the safe ones. The obtained results investigated that apoptosis was one of the main mechanisms of compounds 1, 3c to inhibits cancer cell proliferation (Fig. 5).

#### 3.2.5. Antimicrobial activity

It has been employed that various pyrazole and thiazole motifs constitute the main blocks in various promising antimicrobial compounds.^64,65^ Thus, it was interest to assess the antimicrobial activity of the newly prepared compounds alongside their cytotoxic evaluation as an effort to gain new candidates of dual potent anticancer and antimicrobial activities. The antimicrobial potency of the new compounds 1–5 was *in vitro* screened *versus* a panel of pathogenic microbes: Gram-positive bacteria *viz. S. aureus*, *E. faecalis*, Gram-negative bacteria *viz. S. pneumoniae*, *E. coli*, *S. typhi* and two fungal and yeast strains *viz.* A. *fumigatus*, *C. albicans* using streptomycin and amphotericin B as reference drugs for antibacterial and antifungal activity, respectively. Agar well diffusion approach was used to examine the antimicrobial activity of all synthesized compounds and the obtained data for each examined compound were recorded as the mean diameter of inhibition zones (mm) for the bacterial or fungal growth around the discs^67^ (Table 3). Moreover, two-fold serial dilution approach was used to find out the Minimum Inhibitory Concentration (MIC) values (expressed in μg mL^−1^) for the examined compounds.^68,69^ The obtained data were recorded in (Table 4).

**Table 3:** *In vitro* antimicrobial activities of the synthesized compounds using well diffusion agar assay and expressed as mean diameter of inhibition zones (mm)

Mean diameter of inhibition zone (mean ± SEM) (mm)								
Cpd. no.	Gram +ve bacteria			Gram −ve bacteria			Fungi	
	*S. aureus* 25923	*E. faecalis*	*S. pneumoniae* 010010	*E. coli* RCMB 010052	*S. typhi* ATCC 14028	*P. aeruginosa*	*A. fumigatus* RCMB 02568	*C. albicans* ATCC 10231
1	31 ± 0.17	28 ± 0.92	30 ± 0.87	26 ± 0.22	21 ± 0.54	27 ± 0.51	25 ± 0.88	22 ± 0.30
3a	28 ± 0.15	13 ± 0.76	21 ± 0.69	25 ± 0.15	17 ± 0.95	22 ± 0.44	18 ± 0.25	12 ± 0.51
3b	19 ± 0.68	20 ± 0.24	14 ± 0.84	15 ± 0.71	23 ± 0.79	17 ± 0.41	21 ± 0.22	11 ± 0.70
3c	30 ± 0.47	27 ± 0.36	25 ± 0.17	28 ± 0.92	21 ± 0.16	23 ± 0.13	24 ± 0.15	18 ± 0.67
3d	13 ± 0.95	22 ± 0.11	28 ± 0.45	22 ± 0.14	18 ± 0.13	20 ± 0.19	19 ± 0.45	16 ± 0.28
5a	21.2 ± 0.25	13 ± 0.43	NA	NA	23 ± 0.05	NA	9 ± 0.53	NA
5b	16 ± 0.88	27 ± 0.41	28 ± 0.12	10 ± 0.42	NA	18 ± 0.95	NA	12 ± 0.07
5c	20 ± 0.08	24 ± 0.89	15 ± 0.86	25 ± 0.10	17 ± 0.78	22 ± 0.48	26 ± 0.17	19 ± 0.23
5d	25 ± 0.13	NA	NA	15 ± 0.04	NA	13 ± 0.11	NA	NA
aS	26 ± 0.45	25 ± 0.38	22 ± 0.81	27 ± 0.56	23 ± 0.92	20 ± 0.53	—	—
aA	—	—	—	—	—	—	22 ± 0.72	20 ± 0.78

a Streptomycin and Amphotericin B were used as standard drugs against the tested bacteria and fungi, respectively.

**Table 4:** Minimal inhibitory concentrations (MIC) (μM) of the target compounds against the tested pathogenic bacteria and fungi

	Gram +ve bacteria			Gram −ve bacteria			Fungi	
Cpd. no.	*S. aureus* 25923	*E. faecalis*	*S. pneumoniae* 010010	*E. coli* RCMB 010052	*S. typhi* ATCC 14028	*P. aeruginosa*	*A. fumigatus* RCMB 02568	*C. albicans* ATCC 10231
1	3.63	16.14	3.94	18.33	62.18	7.52	20.81	38.26
3a	16.85	185.18	58.86	19.36	125.14	40.45	120.05	186.11
3b	78.98	67.41	178.52	118.98	45.62	124.84	62.12	189.05
3c	4.12	17.28	20.15	14.18	64.05	42.18	19.23	18.41
3d	181.12	75.47	16.18	71.82	121.43	67.02	117.96	132.26
5a	63.57	183.27	—	—	44.68	—	197.41	—
5b	135.36	15.18	15.87	193.20	—	121.75	—	185.13
5c	68.75	18.86	178.06	20.75	125.36	62.25	17.83	79.42
5d	18.87	—	—	118.15	—	180.25	—	—
aaS	16.75	19.48	65.33	9.18	47.12	68.25	—	—
aaA	—	—	—	—	—	—	39.15	67.82

a Streptomycin and amphotericin B were used as standard drugs against the tested bacteria and fungi, respectively.

Based on the MIC records in Table 4, it has been detected that there is a wide variability in the antimicrobial potency of the newly tested members. Evidently, the pyrazolinyl-aminothiazol-one 1 and the thieno[3,2-*d*]thiazole-carbohydrazide derivative 3c produced the most potent broad spectrum antimicrobial activity against the test bacterial and fungal strains.

It has been detected that the compounds 1 and 3c exhibited selective antibacterial activity against the tested Gram-positive bacteria of 1.20–16 folds more potency than streptomycin of MIC range; 3.63–20.15 μM, MIC_streptomycin_; 16.75–65.33 μM. Conversely, the potency of the latter compounds decreased by 2 and 1.3 folds against the Gram-negative bacteria *S. pneumoniae* and *E. coli* of MIC range; 14.18–64.05 μM, MIC_streptomycin_; 9.18, 47.12 μM. On the other hand, *P. aeruginosa* strain represented greater selectivity towards 1, 3c than streptomycin of MIC values; 7.52, 42.18 μM, respectively, MIC_streptomycin_; 68.25 μM. In addition, the fungal and yeast strains under study exhibited 1.8–3.6 times more sensitivity towards the compounds 1 and 3c than the reference drug amphotericin B producing MIC range 18.41–38.26 μM, MIC_amphotericin B_; 68.25 μM. Furthermore, the thieno [3,2-*d*]thiazole-6-carbonitrile analogue 3a produced equipotent antibacterial activity to streptomycin against *S. aureus* and 1.1, 1.6 folds more potency against *S. pneumoniae* and *P. aeruginosa* of MIC values; 16.85, 58.86 μM. On the other hand, the antifungal activity 3a was completely lost against the tested fungal and yeast strains. Overall, a majority of the target compounds showed moderate to weak activity against the tested kinds of susceptible strains.

The obtained results confirmed that the compounds 1 and 3c among the whole tested derivatives were the most promising antimicrobial agents against the examined Gram-positive bacteria and the fungal strains, but they produced a slightly weaker activity against the examined Gram-negative microbes.

### 3.3. Computational studies

#### 3.3.1. Molecular docking study on EGFR, VEGFR-2 and BRAF^V600E^

The docking simulation was applied in attempt to achieve further vision of the binding modes and orientations of the promising derivatives 1 and 3c into the binding sites of EGFR, VEGFR-2 and BRAF^V600E^ kinases. This study was done using MOE-Dock (Molecular Operating Environment) software version 2014.0901.^70–72^ The docking process was first validated through self-docking of the co-crystallized ligands erlotinib, sorafenib and SB-590885 within the active sites of EGFR, VEGFR-2 and BRAF^V600E^ (PDB codes: 1M17, 4ASD and 2FB8, respectively)^73–75^ giving energy scores of −12.23, −11.50 and −11.37 kcal mol^−1^ with small RMSD values of 0.88, 1.24 and 1.35 Å, respectively between the co-crystallized ligands and their docked poses.

**Fig. 6:**
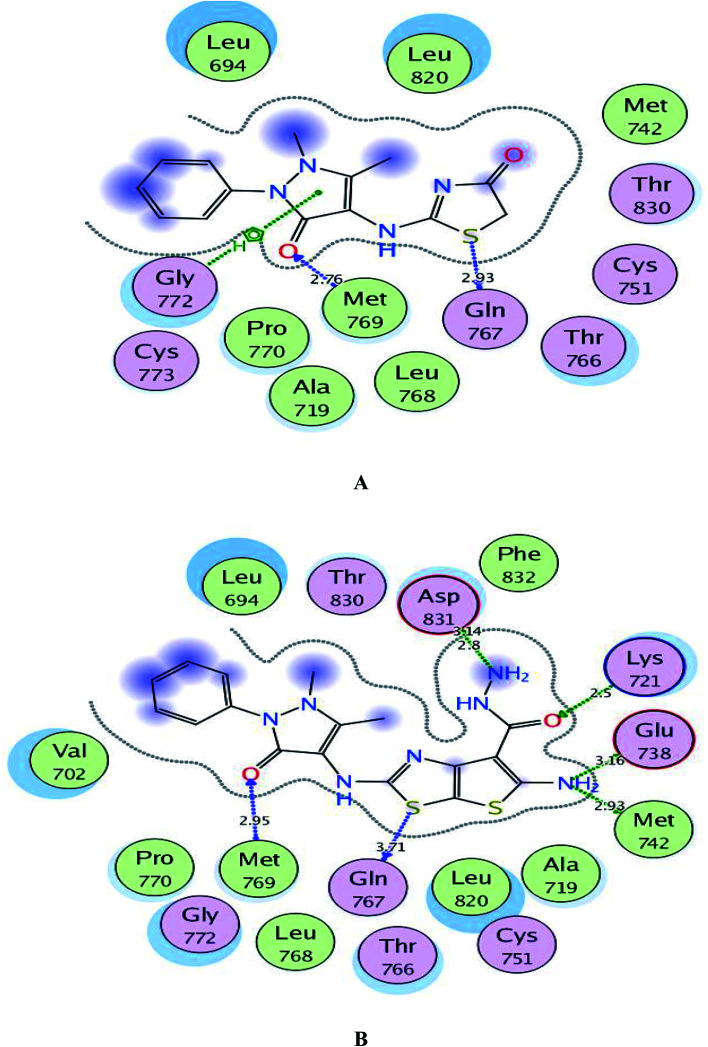
A and B maps illustrate the 2D binding features of the promising targets 1 and 3c within the active site of EGFR (PDB code: 1M17), respectively.

As depicted in Fig. 6, the targets 1 and 3c were bound to the active site of EGFR with energy scores of −11.67 and −11.83 kcal mol^−1^, respectively through formation of two H-bonds, one acceptor between the oxygen of the pyrazolone moiety and the backbone of the key amino acid Met769, and the other one was donor between the thiazolyl sulfur and the backbone of Gln767 (distance: 2.76 & 2.93 Å in compound 1 and 2.95 & 3.71 Å in compound 3c, respectively). Also, the pyrazolone centroid of compound 1 afforded arene-cation interaction with Gln772. On the other hand, the nitrogen of the amino group at p-2 of thienothiazole moiety in compound 3c gave two H-bond donors with the sidechains of Glu738 and Met742 (distance: 3.16 and 2.93 Å, respectively). Also, the carbohydrazide fragment improved fixation of compound 3c through formation of additional H-bonds. One was between the oxygen and the sidechain of Lys721 (distance: 2.50 Å, respectively), and the two others were between the amino nitrogen and the sidechain of Asp831 (distance: 2.80 and 3.14 Å, respectively).

**Fig. 7:**
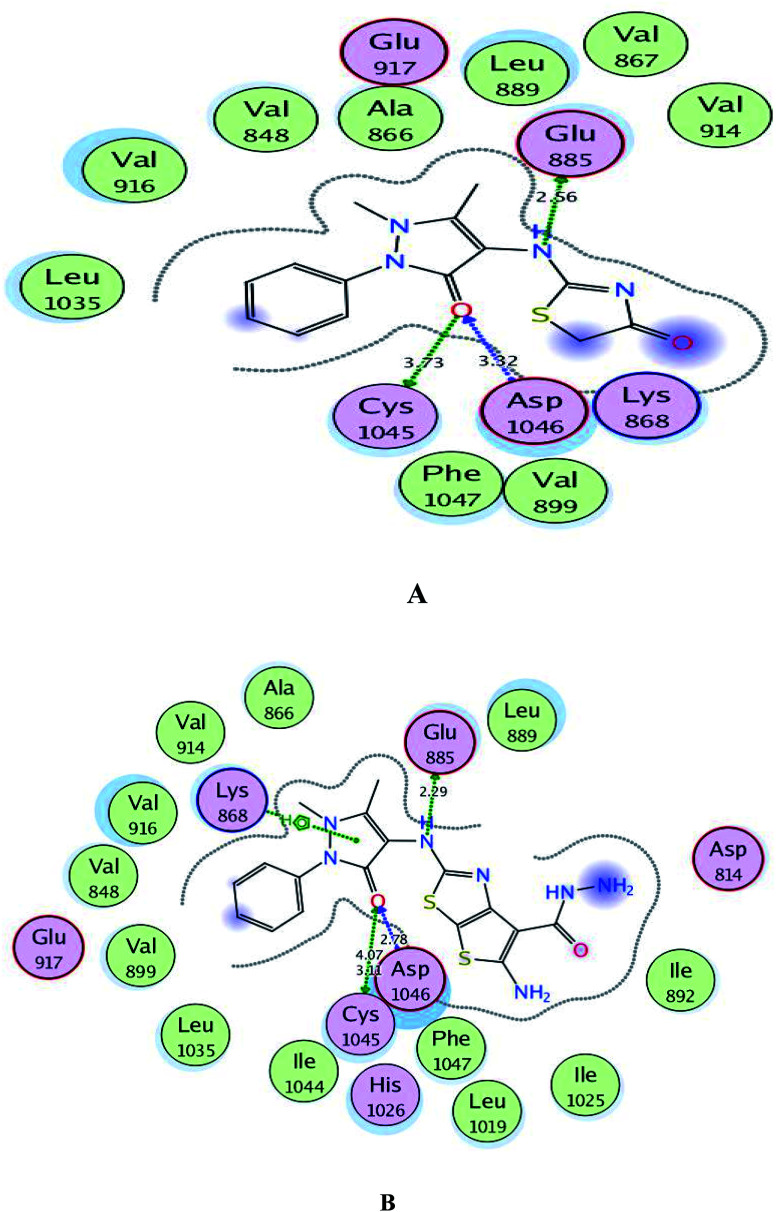
A and B maps illustrate the 2D binding features of the promising targets, 1 and 3c within the active site of VEGFR-2 (PDB code: 4ASD), respectively.

Regarding to binding to VEGFR-2 active site in Fig. 7, it was observed that the pyrazolinone oxygen and NH linker in both compounds played an essential role in the fixation through formation of hydrogen bonding with the amino acids Cys1045, Asp1046 and Glu885. Moreover, the pyrazolinone scaffold in compound 3c displayed arene-cation interaction with Lys868. The targets 1 and 3c provided small energy scores of −10.63 and −11.22 kcal mol^−1^, respectively confirming their stability within the binding pocket.

**Fig. 8:**
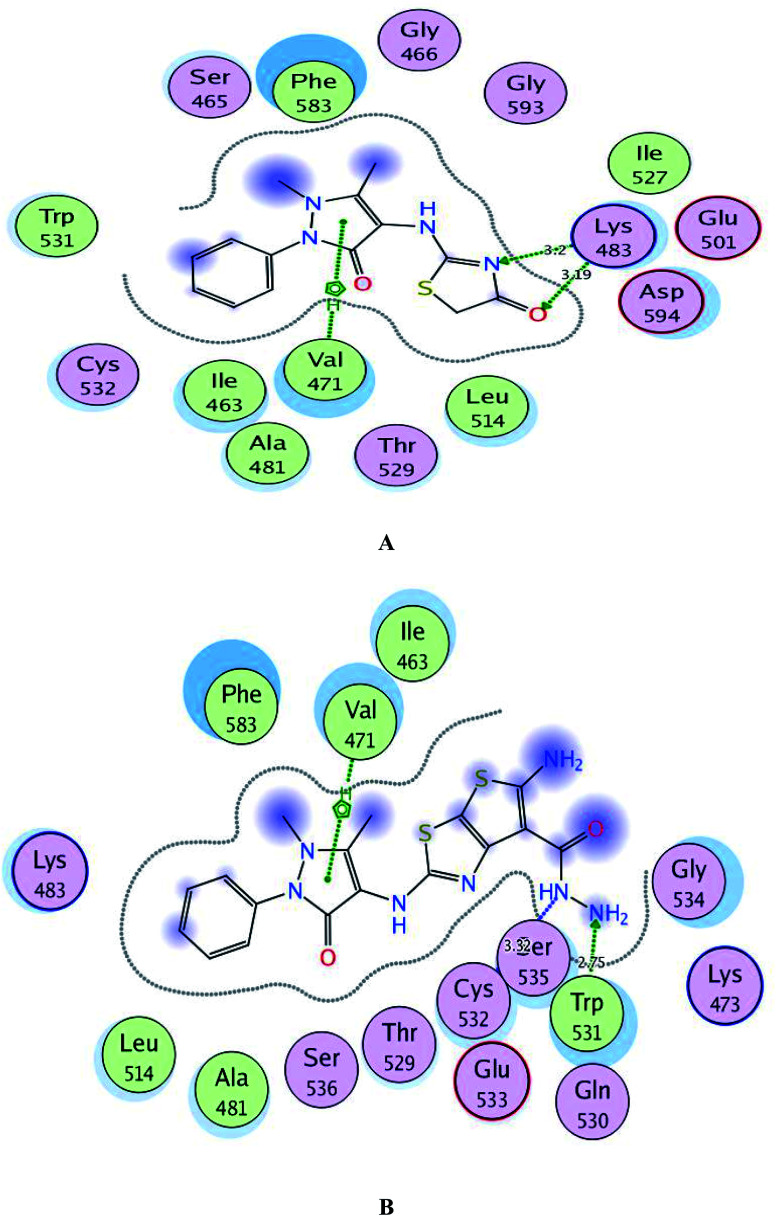
A and B maps illustrate the 2D binding features of the promising targets, 1 and 3c within the active site of BRAF^V600E^ (PDB code: 2FB8), respectively.

By inspection of Fig. 8, the derivatives 1 and 3c were bound to the vicinity of BRAF^V600E^ with energy scores of −10.25 and −10.92 kcal mol^−1^, respectively through arene-cation interaction between the pyrazolinone moiety and the amino acid Val471. Furthermore, the nitrogen and the carbonyl oxygen of thiazolinone scaffold in compound 1 exhibited two H-bond acceptors with the sidechain of Lys483 (distance: 3.20 and 3.19 Å, respectively). While the two nitrogens of the hydrazide fragment in the compound 3c revealed two H-bonds with the sidechain of Trp531 and the backbone of Cys532 (distance: 2.75 and 3.32 Å, respectively).

**Fig. 9:**
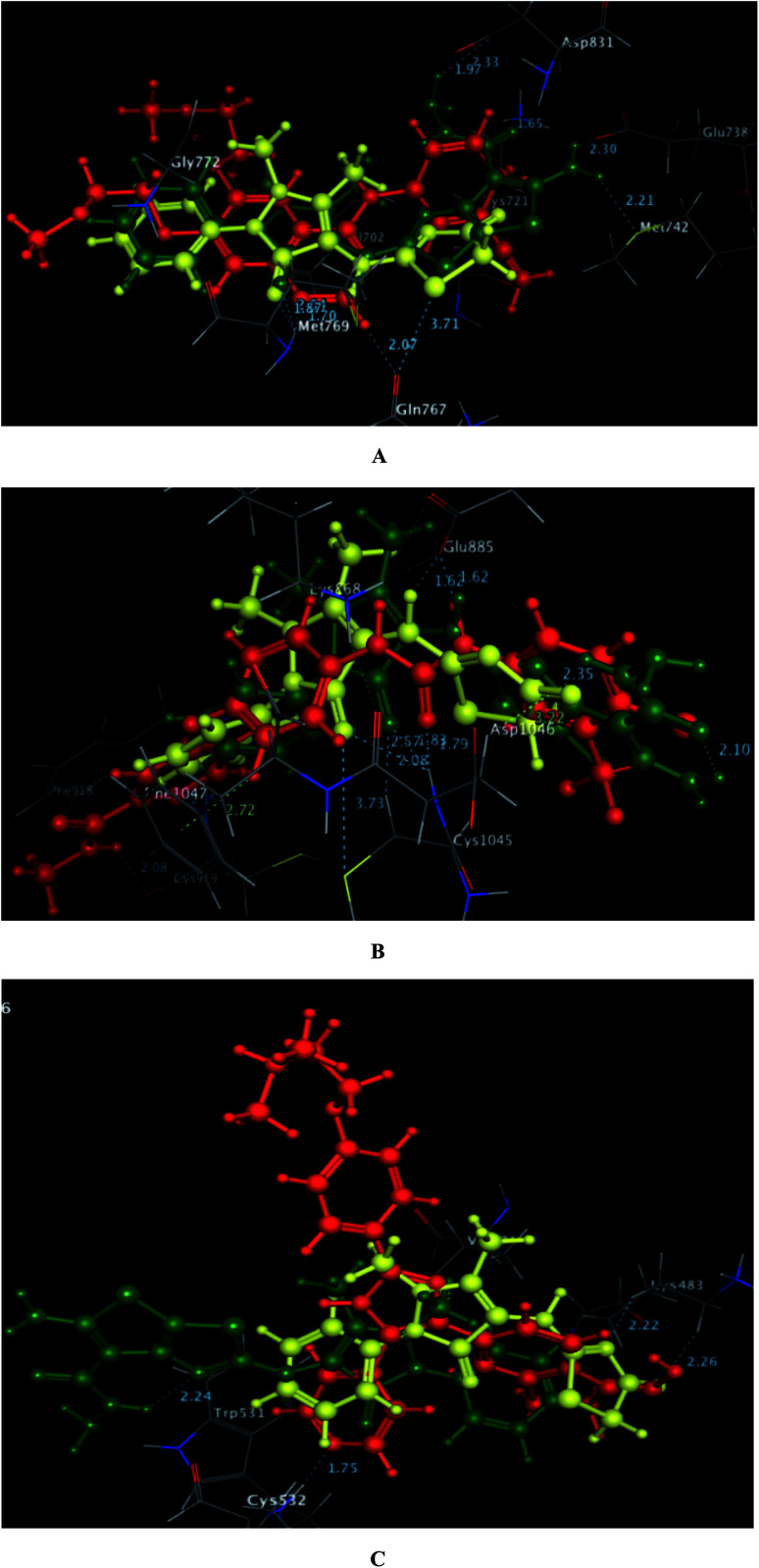
A–C diagrams explore the 3D superimposition between the promising targets, 1 (yellow), 3c (green) and the docked original ligands, erlotinib, sorafenib and SB-590885 (red) within the binding sites of EGFR, VEGFR-2 and BRAF^V600E^ (PDB codes: 1M17, 4ASD and 2FB8, respectively).

Finally and regarding to the superimposition Fig. 9, it was noted that the presence of pyrazolinone scaffold linked to thiazolinone moiety in compound 1 or thieno[3,2-*d*]thiazole-6-carbohydrazide in compound 3c gave the chance for good fitting within the active sites of EGFR, VEGFR-2 and BRAF^V600E^ with a relatively similar binding behavior as the original ligands erlotinib, sorafenib and SB-590885 through arene-cation and H-bond interactions.

#### 3.3.2. *In silico* toxicity potential

The newly synthesized analogues were estimated for toxicity risks and physicochemical characteristics *via* Osiris methodology.^76^ The toxicity parameters including mutagenicity, tumorgenicity, skin irritancy and reproductive effect were predicted for all compounds 1–5 and the results were cited in Table 5. The resultant data showed that all the compounds would be safe and were predicted to have no side effects. Drug score measures the compound's potential to have drug-conform behavior. Accordingly, the highest drug-score value was obtained by compound 1 proving its both effectiveness and potentiality as a new drug member.

**Table 5:** Predictive toxicity of the synthesized compounds according to molinspiration software

Comp. no.	Toxicity risks				Solubility	Drug-likeness	Drug score
	Mutagenicity	Tumorigenicity	Irritancy	Reproductive effect			
1	Red	Red	Green	Green	−2.62	5.3	0.33
3a	Red	Red	Green	Green	−5.83	0.88	0.18
3b	Red	Red	Green	Green	−5.5	1.85	0.19
3c	Red	Red	Green	Green	−5.43	0.05	0.16
3d	Red	Red	Green	Green	−5.08	5.14	0.23
5a	Red	Red	Green	Green	−6.94	5.38	0.16
5b	Red	Red	Green	Green	−5.22	5.8	0.23
5c	Red	Red	Green	Green	−4.92	5.19	0.24
5d	Red	Red	Green	Green	−7.68	5.83	0.14

## 4. Conclusions

This context represents the design and synthesis of two sets of derivatives bearing pyrazoline-3-one ring integrated either with the thieno[3,2-*d*]thiazole or with thiazolo[4,5-*d*]thiazolidine-2-thione scaffolds *via* NH linker, 3a–d, 5a–d, respectively using the pyrazolinyl-thiazolinone derivative 1 as a key precursor. All the new derivatives were evaluated as cytotoxic candidates against two human cancer cell lines; breast MCF-7 and hepatocellular HepG-2, using doxorubicin as a reference drug. Compounds 1 and 3c revealed significant inhibitory activities against both cancer cell lines with IC_50 s_ ranging from 4.02–8.35 μM, comparing to doxorubicin of IC_50 s_; 4.62, 5.66 μM. In addition, compounds 1 and 3c showed a promising safety profile against the normal WI38 cell line. Furthermore, the compounds were evaluated as multitargeting protein kinase inhibitors against EGFR, VEGFR-2 and BRAF^V600E^. Both compounds showed the most potent suppression effect against EGFR of IC_50 s_; 0.022, 0.017 μM, comparing to the reference drug sorafenib of IC_50_; 0.025 μM. The inhibitory activities of 1 and 3c decreased slightly against VEGFR-2 and BRAF^V600E^ of IC_50_ range; 0.040–2.259 μM, IC_50 sorafenib_; 1.022, 0.040 μM. Moreover, compounds 1 and 3c were tested for their impact on cell cycle progression and induction of apoptosis in the MCF-7 cell line. It was investigated that they produce an apoptotic effect and cell cycle arrest. Additionally, all the new analogues were assessed as antibacterial and antifungal agents against a number of pathogenic Gram-positive, Gram-negative bacteria, yeast and fungi in comparison to streptomycin and amphotericin-B. Interestingly, both I and 3c appeared the most potent antimicrobial members against the examined microbial stains considering both compounds having dual anticancer and antimicrobial activities. Molecular docking study rationalized the promising suppression effect of 1 and 3c against EGFR, VEGFR-2 and BRAF^V600E^ due to their good fitting and the binding energy scores in the active site of the tested kinases. Additional toxicity study was performed for all new derivatives which represented their good drug-likeness properties and medium toxicity risks in humans.

## Conflicts of interest

The authors declare that they have no conflict of interest.

## Supplementary Material

RA-012-D1RA08055E-s001
