# Supplementary material for: Synthesis and biological evaluation of new derivatives of thieno-thiazole and dihydrothiazolo-thiazole scaffolds integrated with a pyrazoline nucleus as anticancer and multi-targeting kinase inhibitors
Source: RSC Adv. 2021 Dec 22;12(1):561–77. doi: 10.1039/d1ra08055e (PMC8694192; doi:10.1039/d1ra08055e)
Supplement: RA-012-D1RA08055E-s001 [file RA-012-D1RA08055E-s001.pdf]

Electronic Supplementary Material (ESI) for RSC Advances.
This journal is © The Royal Society of Chemistry 2021

Supplementary material

1. Experimental

1.1. Chemistry

Melting points are uncorrected and are determined in open capillary tubes using electric melting point apparatus (G-K). Infrared spectra (KBr discs) were measured on a Shimadzu FTIR, 8300 PC IR spectrophotometer. ^{1}H NMR (400 MHz) and ^{13}CNMR (100 MHz) was recorded with a Bruker model Ultra Shield NMR spectrometer with TMS as the internal standard and chemical shifts were reported on a δ scale (ppm) using DMSO-d_6 as solvents, while the coupling constants (*J* values) are given in Hz. Elemental analyses were determined on a PerkinElmer 240, and the values found were within ±0.4% of the theoretical. All reactions were monitored by TLC on Merck Silica Gel 60F254 and spots were detected using a UV lamp (254 nm). The biological activities were carried out in the Medical Mycology Laboratory of the Regional Center for Mycology and Biotechnology of Al-Azhar University, Cairo, Egypt.

1.2. Biological activity

1.2.1. In vitro anticancer screening

The cell lines were purchased from the American Type Culture collection as follows: liver carcinoma cell line (HepG-2) and breast carcinoma cell line (MCF-7). Cytotoxic activity screening was performed using MTT assay at Regional Center for Mycology and Biotechnology, Al- Azhar University. [1] Exponentially, cells were placed in 10^4 cells/ well for 24 h, and then add fresh medium which containing different concentration of the tested sample. Serial two-fold dilutions of the tested sample were added using a multichannel pipette. Moreover, all cells were cultivated at 37 °C, 5% CO_2 and 95% humidity. Also, incubation of control cells occurred at 37 °C. However, after incubation for 24 h different concentrations of sample (50, 25, 12.5, 6.25, 3.125, 1.56 and 0 μg L^{-1}) were added and continued the incubation for 48 h, then, add the crystal violet solution 1% to each well for 0.5 h to examine viable cells. Rinse the wells using water until no stain. After that, add 30% glacial acetic acid to all wells with shaking plates on Microplate reader (TECAN, Inc.) to measure the absorbance, using a test wavelength of 490 nm. Besides, compare the treated samples with the control cell. The cytotoxicity was estimated by IC_{50} in (μM) the concentration that inhibits 50% of growth of cancer cell.

1.2.2. EGFR, VEGFR-2, BRAF^{V600E} kinase inhibitory assay

The most active cytotoxic compounds **1** and **3c** that showed promising IC_{50} values were further examined for their inhibitory activities against EGFR, VEGFR-2 and $BRAF^{V600E}$. [2-4]

EGFR assay: The master mixture (6 µL 5X Kinase Buffer + 1 µL ATP (500 µM) + 1 µL 50 X PTK substrate + 17 µL water) was prepared then, 25 µL to every well was added. 5 µL of Inhibitor solution of each well labeled as "Test Inhibitor" was added. However, for the "Positive Control" and "Blank", 5 µL of the same solution without inhibitor (Inhibitor buffer) was added. 3 mL of 1X Kinase Buffer by mixing 600 µL of 5X Kinase Buffer with 2400 µL water was prepared. So, 3 mL of 1X Kinase Buffer became sufficient for 100 reactions. To the wells designated as "Blank", 20 µl of 1X Kinase Buffer was added. EGFR enzyme on ice was thawed. Upon first thaw, briefly the tube containing enzyme was spun to recover full content of the tube. The amount of EGFR required for the assay and dilute enzyme to 1 ng/µL with 1X Kinase Buffer was calculated. Moreover, the remaining undiluted enzyme in aliquots was stored at -80°C. The reaction was initiated by adding 20 µL of diluted EGFR enzyme to the wells designated "Positive Control" and "Test Inhibitor Control", after that it was incubated at 30°C for 40 minutes. After the 40 minutes reaction, 50 µL of Kinase-Glo Max reagent was added to each well and the plate was covered with aluminum foil and incubated at room temperature for 15 min. Luminescence was measured using the microplate reader.

VEGFR-2 assay: Also, the effect of the most promising cytotoxic compounds **1** and **3c** on the level of VEGFR-2 in human breast cancer cell line MCF-7 was determined. The cells in culture medium were treated with 20 µl of IC50 values of the compounds dissolved in DMSO, then incubated for 24 hours at 37 ºC, in a humidified 5% CO_2 atmosphere. The cells were harvested and the homogenates were prepared in saline using a tight pestle homogenizer until complete cell disruption. The kit uses a double-antibody sandwich enzyme-linked immunosorbent assay (ELISA) to determine the level of human VEGFR-2 in samples. A monoclonal antibody for VEGFR-2 was pre-coated onto 96-well plates. The test samples are added to the wells and a biotinylated detection polyclonal antibody from goat specific for VEGFR-2 was added subsequently followed by washing with PBS buffer. Avidin-Biotin-Peroxidase complex was added and the unbound conjugates were washed away with PBS buffer. HRP substrate TMB was used to visualize HRP enzymatic reaction. TMB was catalyzed by HRP to produce a blue color product that changed into yellow after adding acidic stop solution. The density of yellow color is proportional to the human VEGFR-2 amount of the sample captured in

the plate. The chroma of color and the concentration of the human VEGFR-2 of the samples were positively correlated and the optical density was determined at 450 nm. The level of human VEGFR-2 in samples was calculated (pg/ml) as duplicate determinations from the standard curve. Percent inhibition was calculated in comparison to control untreated cells.

$BRAF^{V600E}$ assay: Reaction Biology Corp. Kinase HotSpotSM service was used for screening of final compounds. Assay protocol: as reported on Reaction Biology Corp. website using 1 μM concentration of ATP. Isolated human BRAF (V599E) was used and MEK1 was used as substrate at 1 uM concentration and 1uM ATP concentration (33P labeled ATP was used to produce 33P-Substrate which was a measure for enzyme activity).

1.2.3. In-vitro DNA-Flow cytometric (cell cycle) analysis

To determine the distribution of cell lines in each phase of cell cycle, the PI was used to stain the DNA content of each cell line. At a density of 1×10^6–3×10^6 cells/dish, MCF-7 cells were seeded in 30 mm tissue culture plates in 5 ml of complete medium. Cells were incubated and allowed to adhere in CO_2 atmosphere. After 24 h adherence, cells were incubated with compound 13k for 24 h. Then, the cell pellets were collected by trypsinization and washed twice with PBS washing buffer and fixed with 70% ice cold ethanol for a minimum of 24 h at −20 °C. The cells were stained with PI and RNase Staining Solution according to the manufacturer's instructions. Cell-cycle distribution was evaluated using a BD FACSCalibur flow cytometer. Data were collected from three individual experiments. [4]

1.2.4. Annexin V-FITC apoptosis assay

Annexin V-FITC apoptosis detection kit (BD biosciences) was used to quantify the percentage of cells undergoing apoptosis and to determine the mode of cell death whether by apoptosis or necrosis in the presence or absence of the active compounds **1** and **3c**. The experiment was carried out according to the manufacturer’s protocol. Briefly, cells were seeded (1×10^6–3×10^6) per dish and allowed to adhere overnight in CO_2 incubator. Following 24 h incubation, the tested compound was added, and plates were incubated for another 24 h in CO_2 atmosphere. Both adherent and nonadherent cells were trypsinized, collected and centrifuged for 5 min at 300g. Cell pellets were washed with 2 ml of cold PBS twice, re-suspended in 100 μl of 1X binding buffer and stained with 5 μl of FITC Annexin V and 5 μl of PI for 15 min in the dark at room temperature. Following incubation, 1 ml of 1X binding buffer was added and the

analysis was done using flow cytometer within an hour. Data was collected from three individual Experiments. [4]

1.2.5. Antimicrobial activity assay

In vitro microbial activities were carryout at the Regional Center for Mycology and Biotechnology (RCMB), Al-Azhar University, Cairo, Egypt. The biological potential of the newly prepared target structures was inspected toward the examined organisms and expressed as the diameter of the inhibition zones due to the agar plate diffusion technique. [5-8] Also, pathological strains (100µl) was outgrowing in 10 mL of fresh media till they reached a count of nearly 108 cells/ml and 105 cells/mL for bacteria and fungi, respectively. Also, each well (10 mm diameter holes cut in the agar gel) included 1mL of each sample (at 0.5 mg/mL). Whoever, incubation of plates was done for 24 h at 37 ˚C for bacteria and72 h at 27˚C for fungi activity. The plates were done in triplicate and the average inhibition zone diameters were recorded in mm and used as criterion for the microbial activity. Tetracycline (standard drug) was also inspected for antibacterial while amphotericin B for the antifungal activity. DMSO (solvent controls) was used for dissolving the examined compounds and illustrated no inhibition zone, indicating that it has no effect on the growth of the tested biological strains. Furthermore, the proper target compounds were further tested to estimate their antimicrobial activity represented as minimum inhibitory concentration (MIC) using the modified agar well diffusion method.

Minimal Inhibitory Concentration (MIC) Measurement

The bacteriostatic activity of the compounds was then evaluated using the two-fold serial dilution technique. Two-fold serial dilutions of the tested compounds solutions were prepared using the proper nutrient broth. The final concentrations of the solutions were 1000, 500, 250, and 125 µg/mL. The tubes were then inoculated with the test organisms, grown in their suitable broth at 37°C for 24 h for the tested microorganisms (1×10^{8} CFU/mL for bacteria and 1 $x10^{6}$ CFU/mLof yeast), each 5 mL received 0.1 mL of the above inoculum and incubated at 37°C for 24 h. The lowest concentration showing no growth was taken as the minimum inhibitory concentration (MIC). [5-8]

1.3. Computational studies

1.3.1. Molecular modeling studies

The 2D structure of the newly synthesized derivatives **1** and **3c** was drawn through chem. Draw. The protonated 3D was employed using standard bond lengths and angles, using

Molecular Operating Environment (MOE-Dock) software version 2014.0901. [9,10] Then, the geometry optimization and energy minimization were applied to get the Conf Search module in MOE, followed by saving of the moe file for upcoming docking process. The co-crystallized structures of EGFR, VEGFR-2 and $BRAF^{V600E}$ kinases with their ligands erlotinib, sorafenib and SB-590885 were downloaded (PDB codes: 1M17, 4ASD and 2FB8, respectively) from protein data bank. [11-13] All minimizations were performed using MOE until an RMSD gradient of 0.05 $kcal{\cdot}mol^{-1}Å^{-1}$ with MMFF94x force field and the partial charges were automatically calculated. Preparation of the enzyme structures was done for molecular docking using Protonate 3D protocol with the default options in MOE. London dG scoring function and Triangle Matcher placement method were used in the docking protocol. At the first, validation of the docking processes were established by docking of the native ligands, followed by docking of the derivatives **1** and **3c** within the ATP-binding sites after elimination of the co-crystallized ligands.

1.3.2. *In silico toxicity potential*

Molecular descriptors display the pharmacokinetic, pharmacodynamics and physicochemical effects of all synthesized targets **1-5**. The lipophilicity (milogP) and topological polar surface area (tPSA) were calculated using the online software Molinspiration, while the aqueous solubility, drug-likeness, drug score were calculated using the OSIRIS property explorer software. Furthermore, according to Veber *et al.*, good bioavailability is more favorable for targets having TPSA of ≤ 140 A°2 and ≤ 10 rotatable bonds. Decreased molecular flexibility, as determined by the rotatable bond number, and low polar surface area or total hydrogen bond count, which are vital predictors of good oral bioavailability, independent of molecular weight.[14]

References

1 A.E. Amr, R.E.A. Mageid, M. El-Naggar, A.M. Naglah and E.S. Nossier and E.A. Elsayed, *Molecules*, 2020, **25**(5), 1096.

2 E.A. Abd El-Meguid, G.O. Moustafa, H.M. Awad, E.R. Zaki and E.S. Nossier, *J. Mol. Struct.*, 2021, **1240**, 130595.

3 L.F. Brown, B. Berse, R.W. Jackman, K. Tognazzi, A.J. Guidi, H.F. Dvorak, D.R. Senger, J.L. Connolly and S.J. Schnitt, *Hum. Pathol.*, 1995, **26**, 86-91.

4 A.S. Hassan, G.O. Moustafa, H.M. Awad, E.S. Nossier and M.F. Mady, *ACS Omega*, 2021, **6**(18), 12361-12374.

5 I. Vermes, C. Haanen, H. Steffens-Nakken and C. Reutellingsperger, *J. Immunol. Methods*, 1995, **184**, 39-51.

6 A.C. Scott, “Laboratory control of antimicrobial therapy,” in Mackie and MacCartney Practical Medical Microbiology, J. G. Collee, J. P. Duguid, A. G. Fraser, and B. P. Marmion, Eds., vol. 2, pp. 161–181, Churchill Livingstone, Edinburgh, Scotland, 13th edition, 1989.

7 Y.M. Syam, M.M. Anwar, E.R. Kotb, S.A. Elseginy, H.M. Awad and G.E. Awad, *Mini Rev. Med. Chem*., 2019, **19**, 1255-1275.

8 H.E. Hashem, A.E. Amr, E.S. Nossier, E.A. Elsayed and E.M. Azmy, *Molecules*, 2020, **25**, 2766.

9 E.S. Nossier, S.M. El-Hallouty and E.R. Zaki, *Int. J. Pharm. Sci*., 2015, **7**, 353-359.

10 S.S. Abd El-Karim, H.S. Mohamed, M.F. Abdelhameed, A.E. Amr, A.A. Almehizia and E.S. Nossier, *Bioorg. Chem*., 2021, **111**, 104827.

11 E.A. Abd El-Meguid, G.O. Moustafa, H.M. Awad, E.R. Zaki and E.S. Nossier, *J. Mol. Struct*., 2021, **1240**, 130595.

12 A.E. Amr, E.A. Elsayed, M.A. Al-Omar, H.O. Badr Eldin, E.S. Nossier and M.M. Abdallah, *Molecules*, 2019, **24**, 416.

13 A.J. King, D.R. Patrick, R.S. Batorsky, M.L. Ho, H.T. Do, S.Y. Zhang, R. Kumar, D.W. Rusnak, A.K. Takle, D.M. Wilson and E. Hugger, *Cancer Res.*, 2006, **66**, 11100-11105.

14 The OSIRIS property explorer software. Available from: http://www.organic-chem istry.org/prog/peo/.

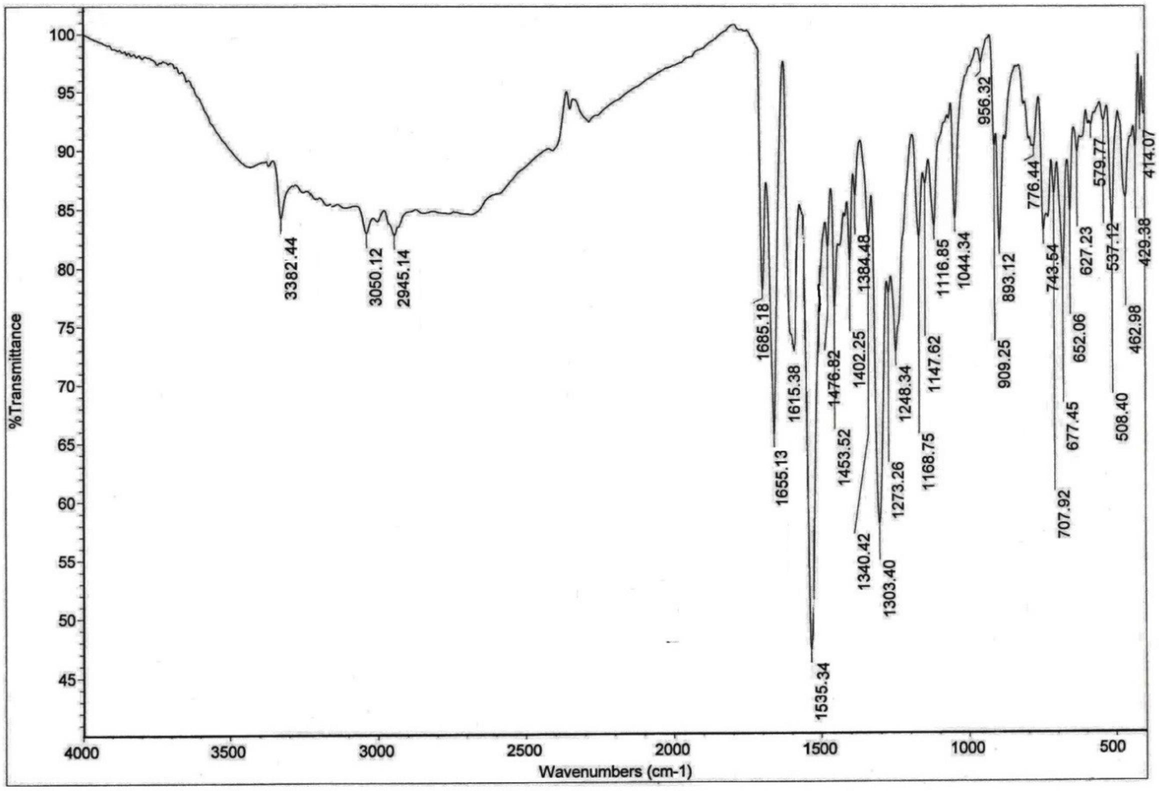

Figure S1. IR spectrum of compound **1**

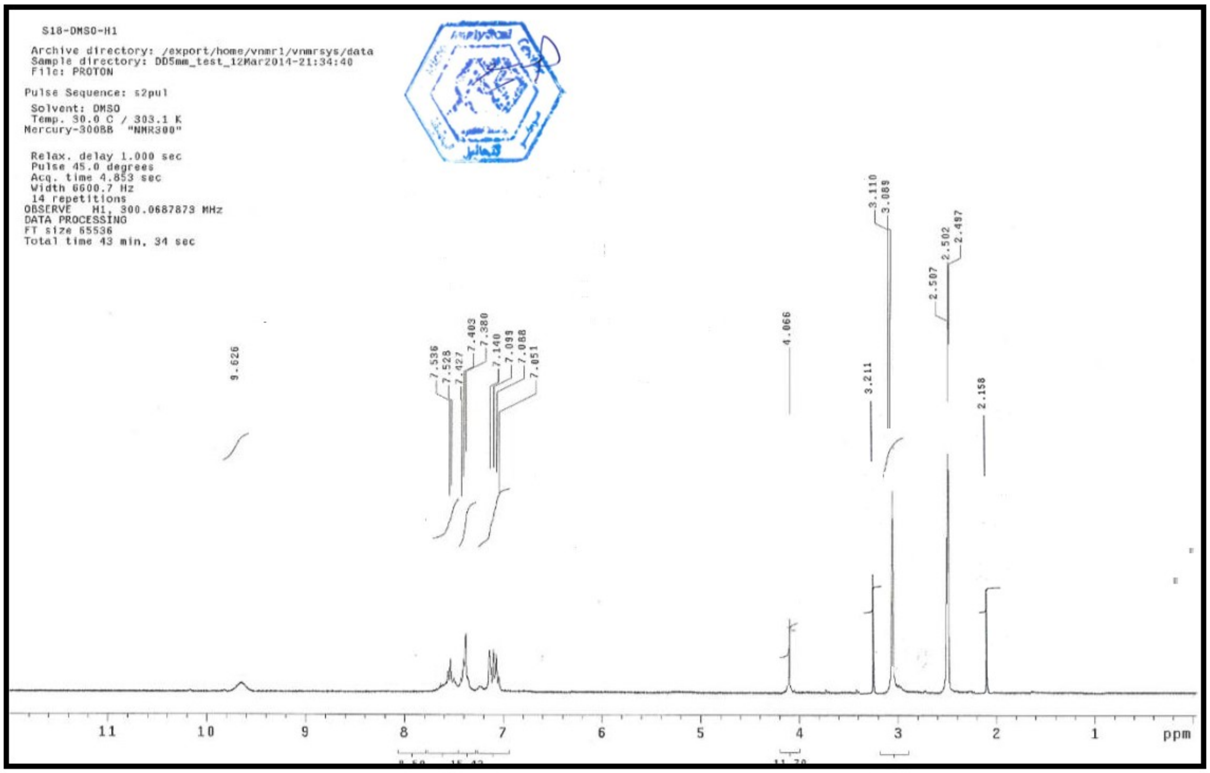

Figure S2. ^{1}H-NMR spectrum of compound **1**

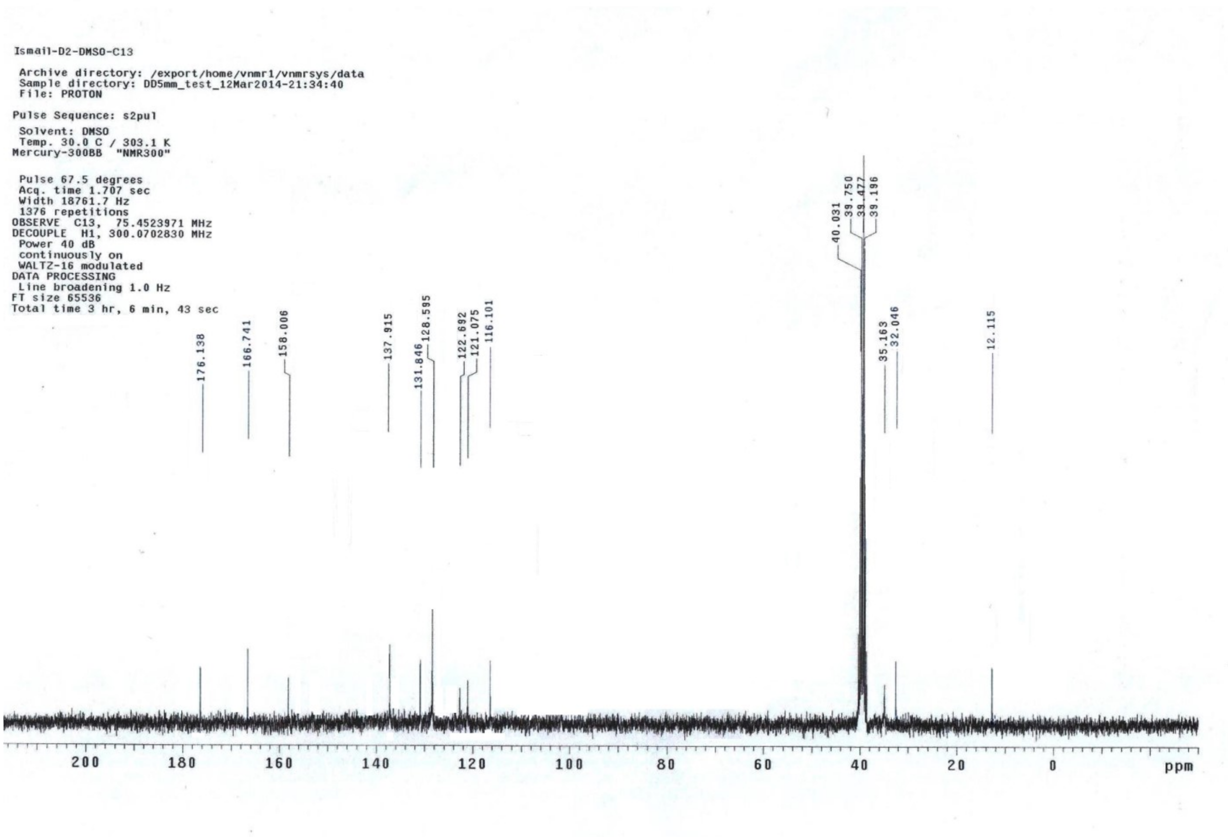

Figure S3. ^{13}C-NMR spectrum of compound **1**

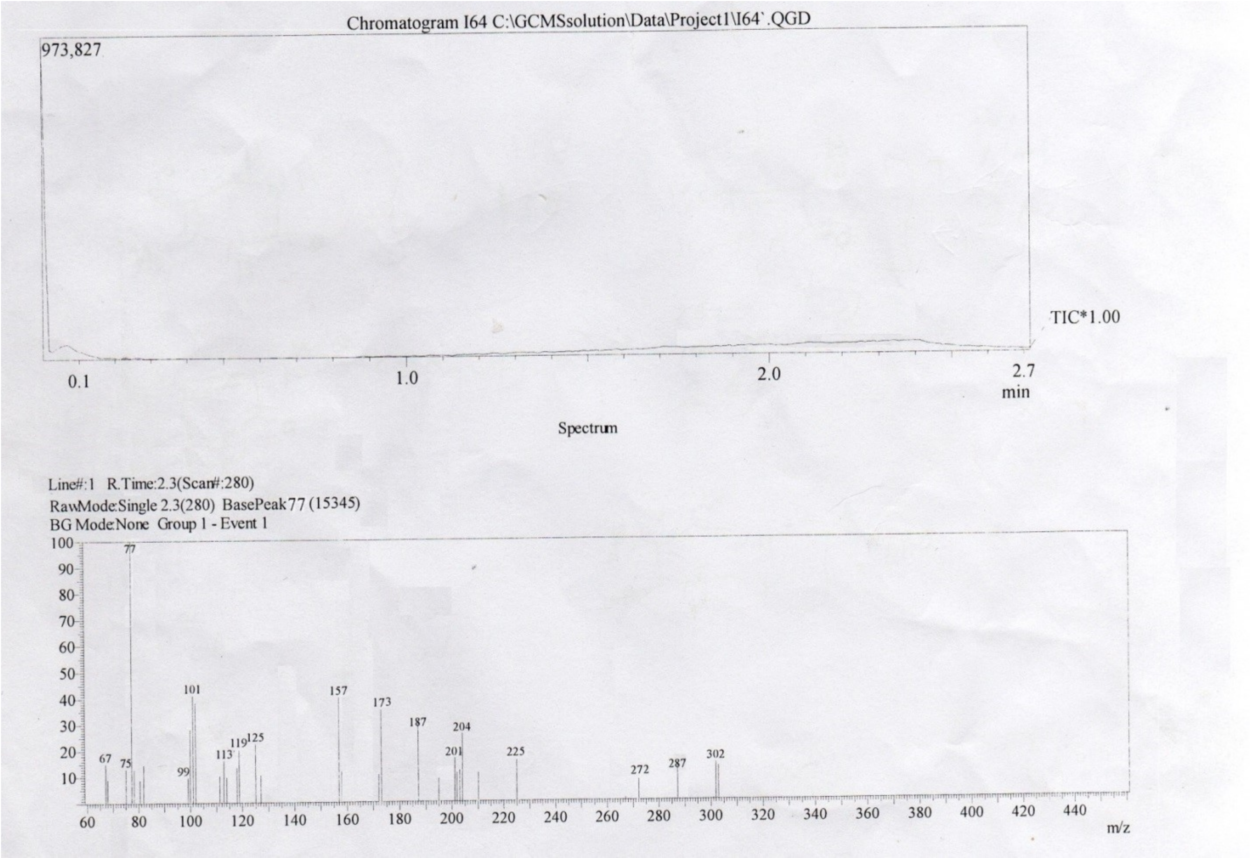

Figure S4. Mass spectrum of compound **1**

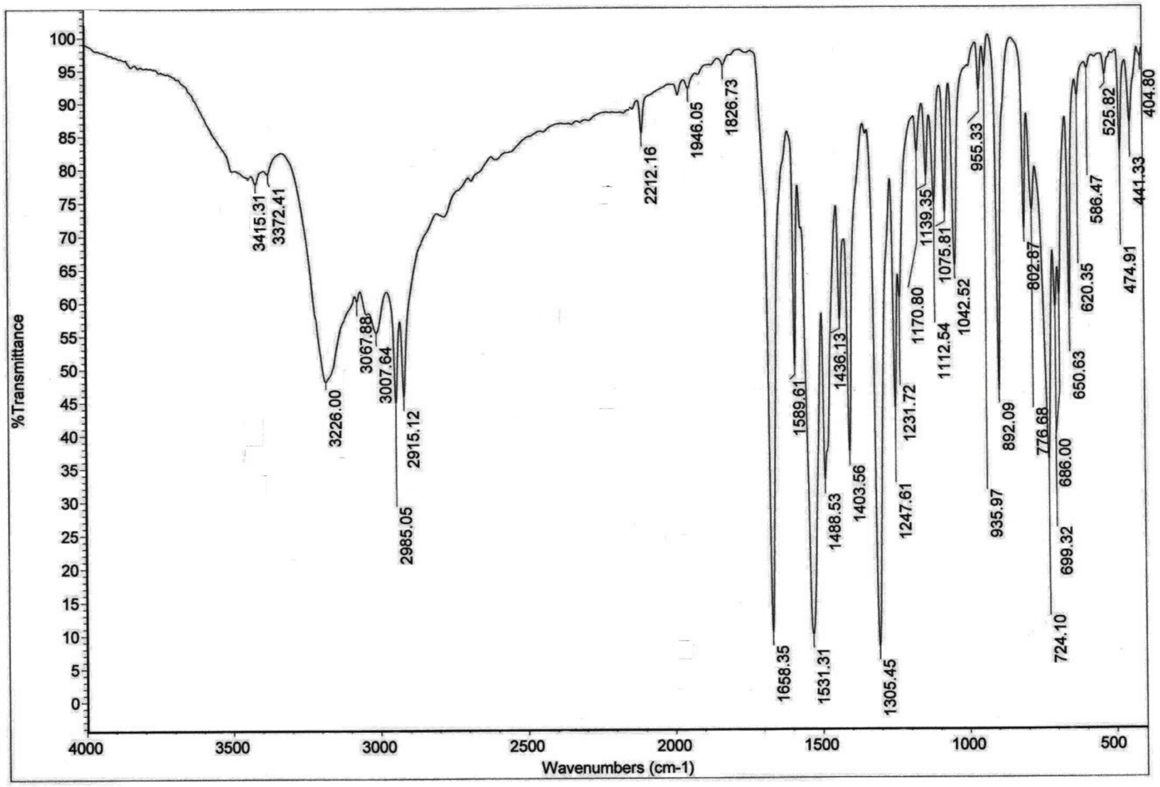

Figure S5. IR spectrum of compound **3a**

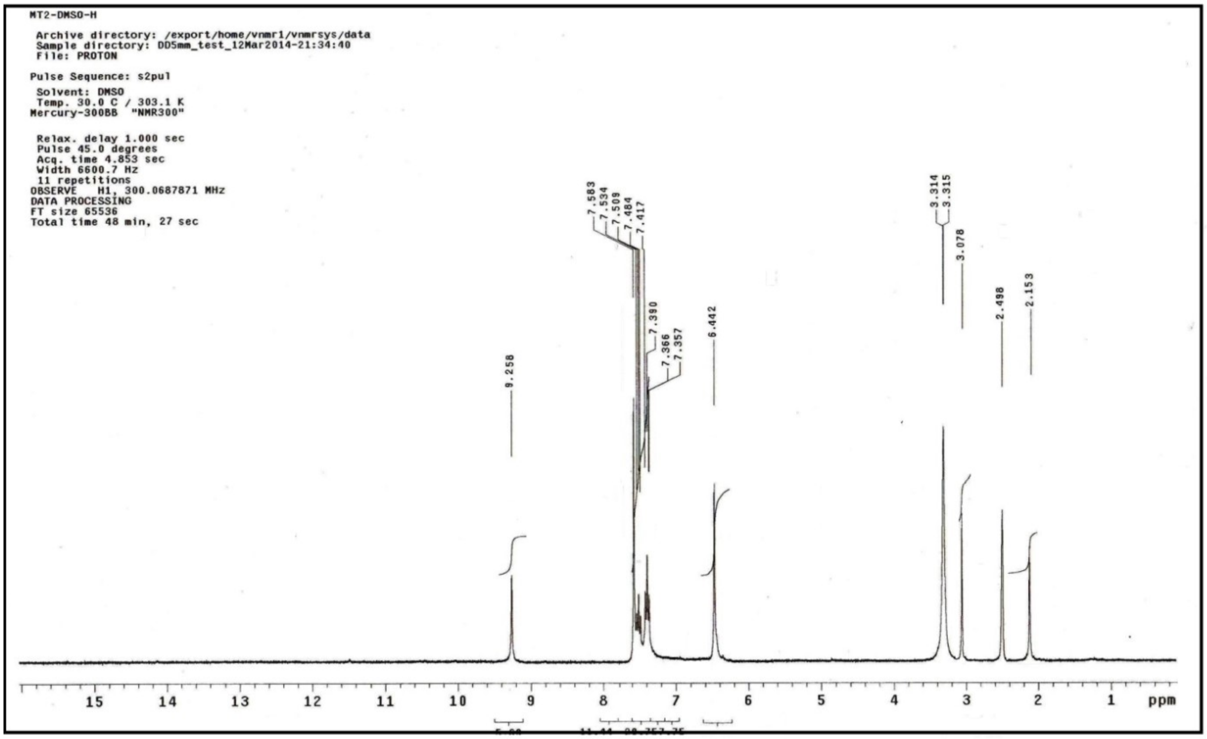

Figure S6. ^{1}H-NMR spectrum of compound **3a**

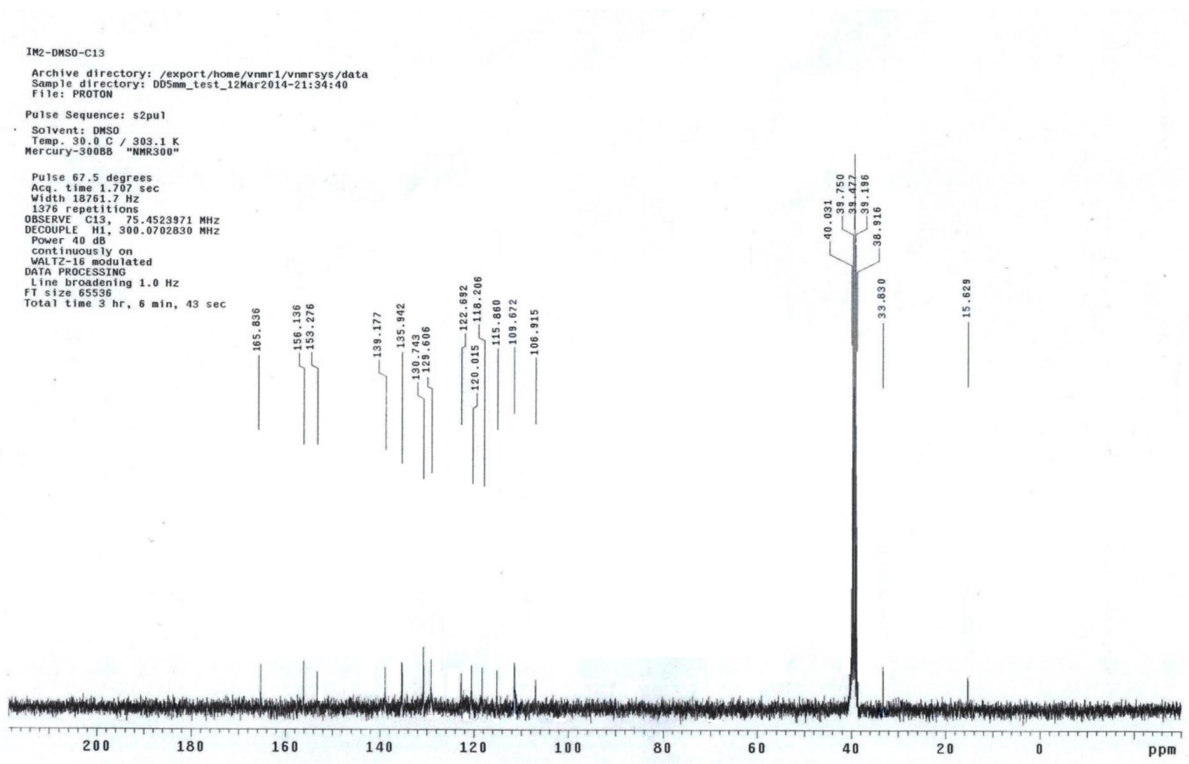

Figure S7. ^{13}C-NMR spectrum of compound **3a**

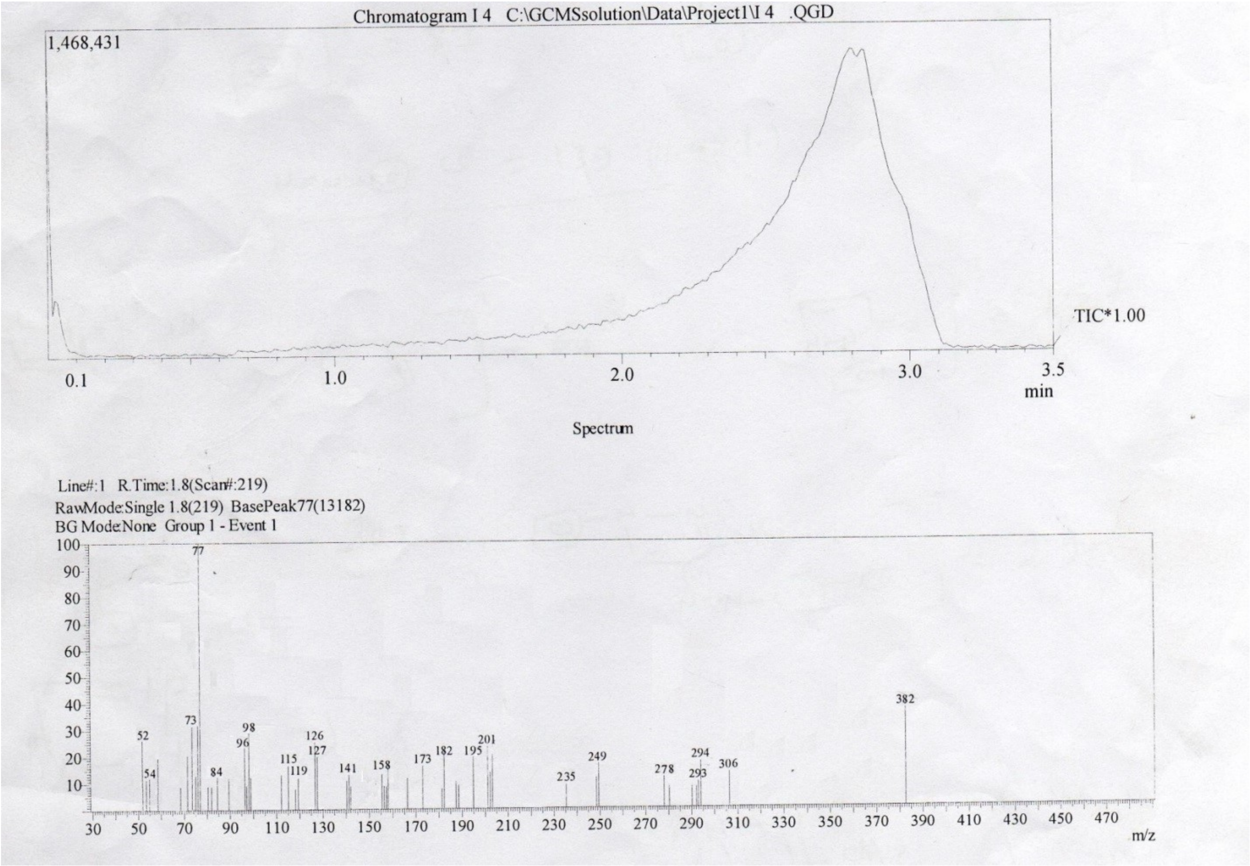

Figure S8. Mass spectrum of compound **3a**

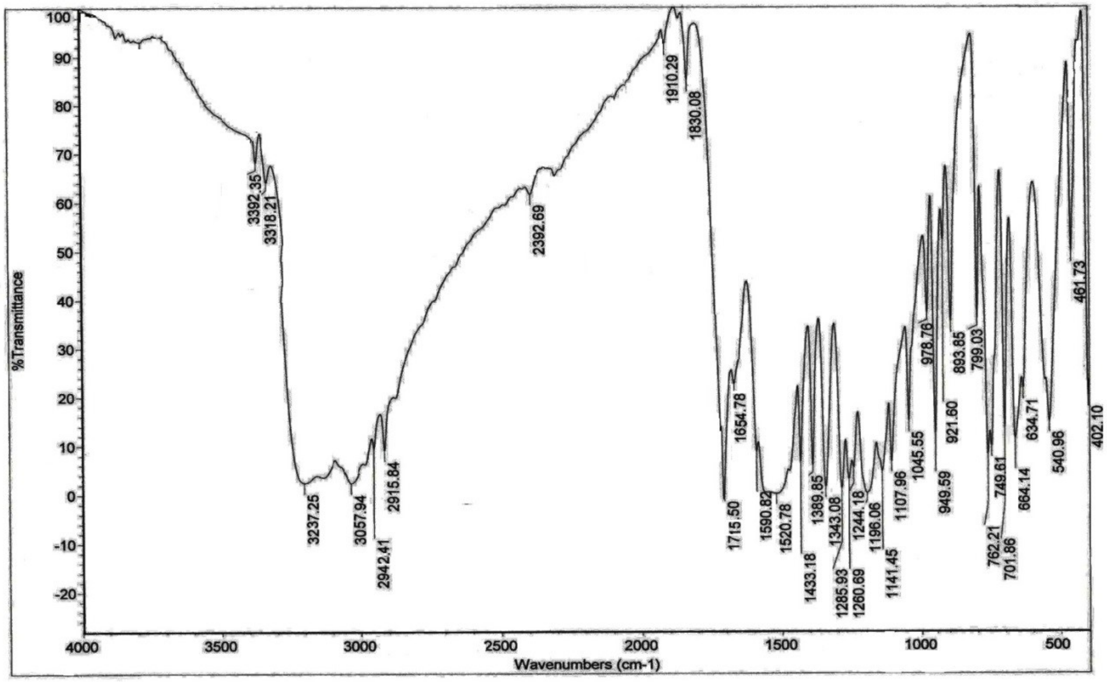

Figure S9. IR spectrum of compound **3b**

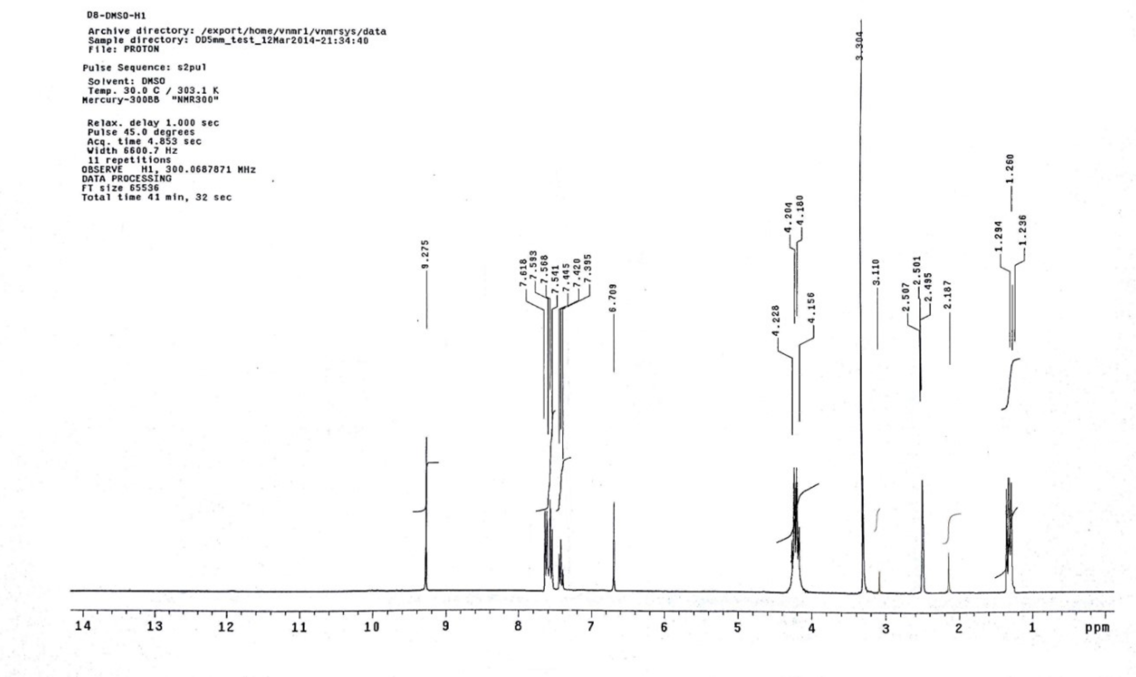

Figure S10. ^{1}H-NMR spectrum of compound **3b**

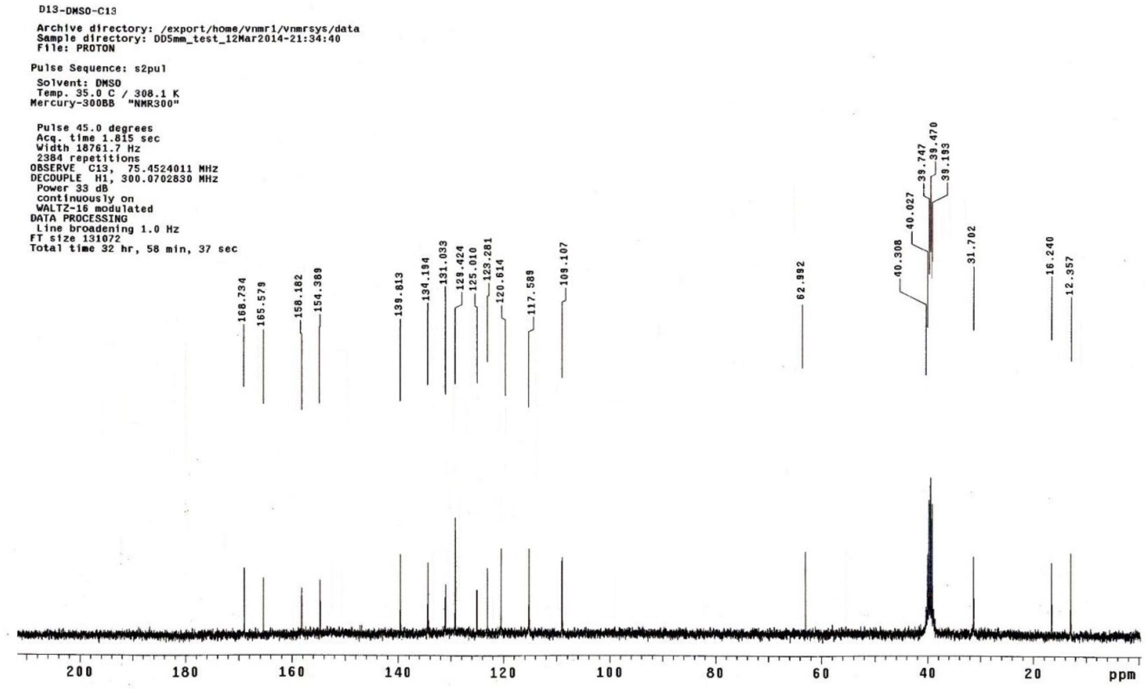

Figure S11. ^{13}C-NMR spectrum of compound **3b**

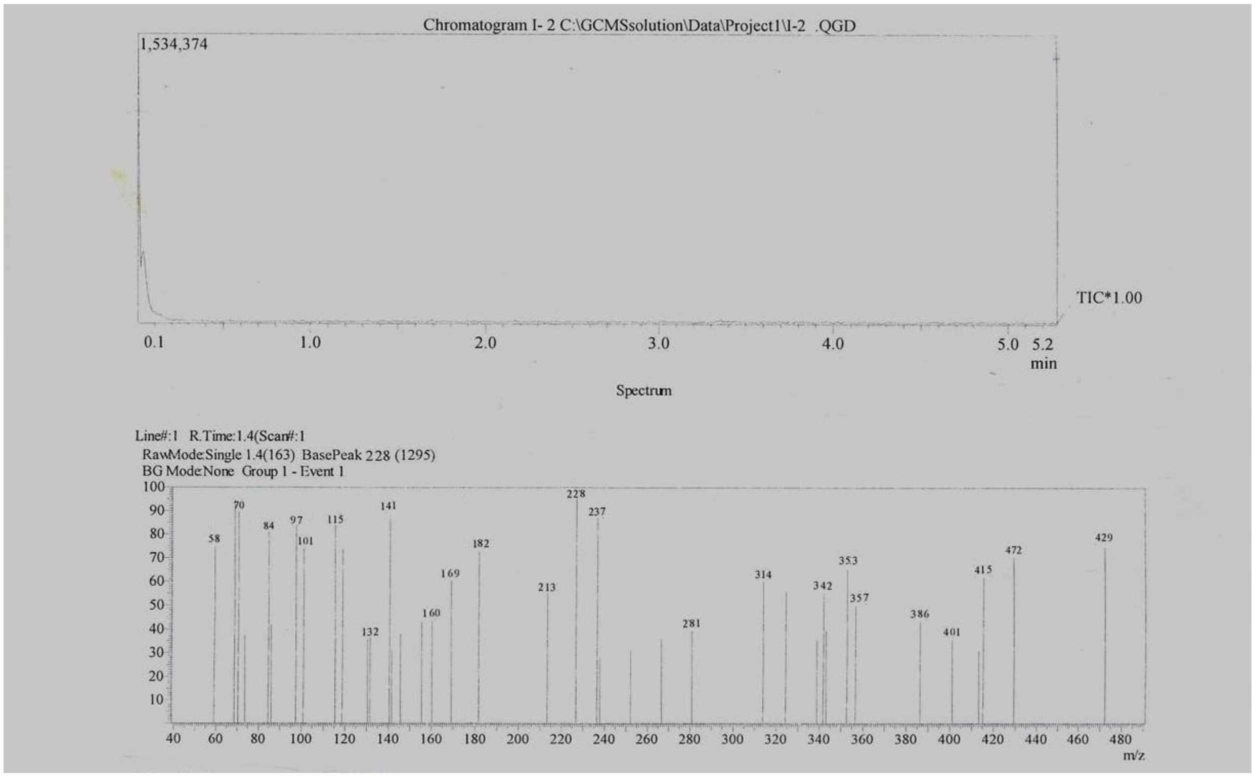

Figure S12. Mass spectrum of compound **3b**

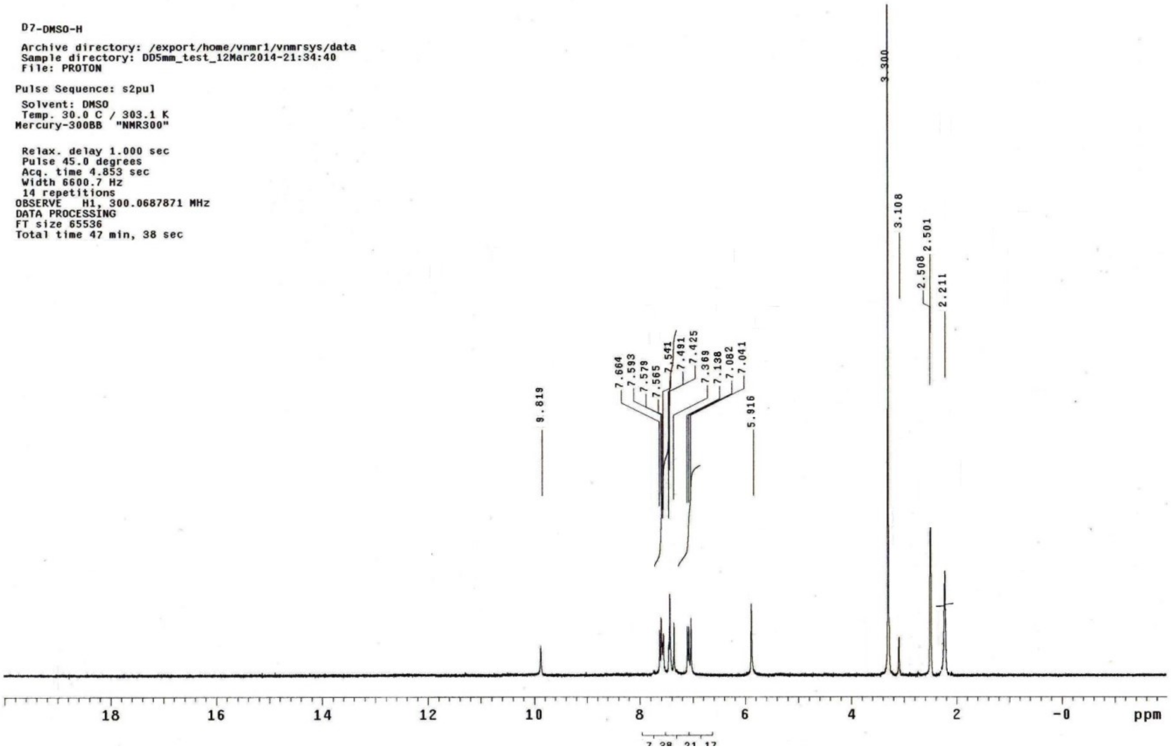

Figure S13. ^{1}H-NMR spectrum of compound **3c**

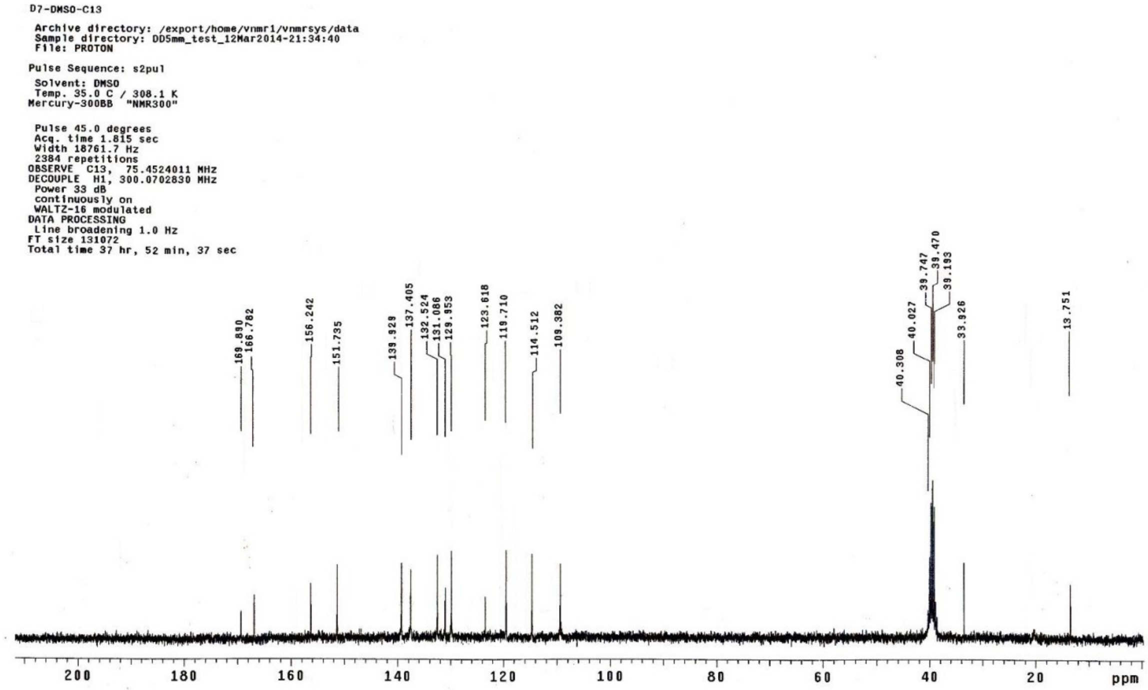

Figure S14. ^{13}C-NMR spectrum of compound **3c**

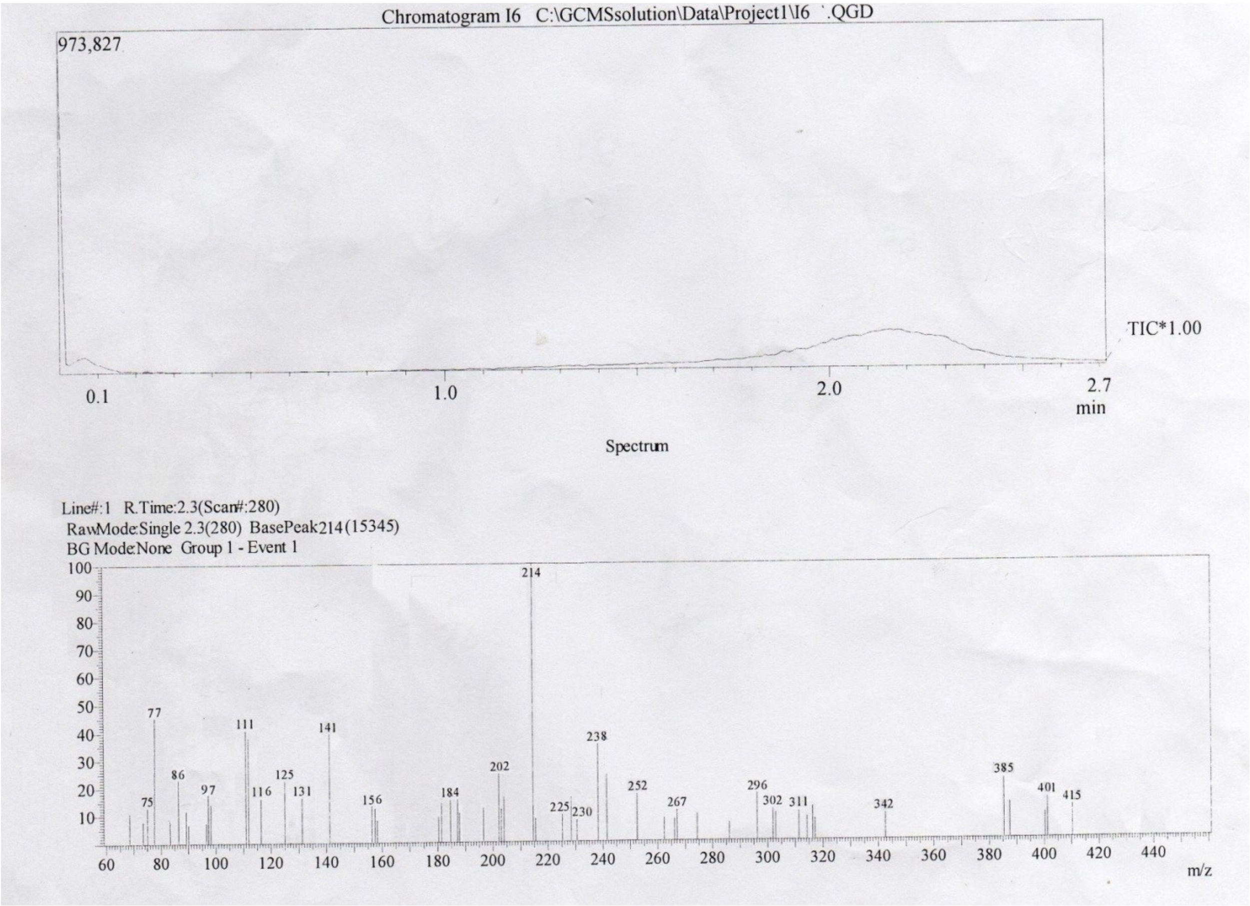

Figure S15. Mass spectrum of compound **3c**

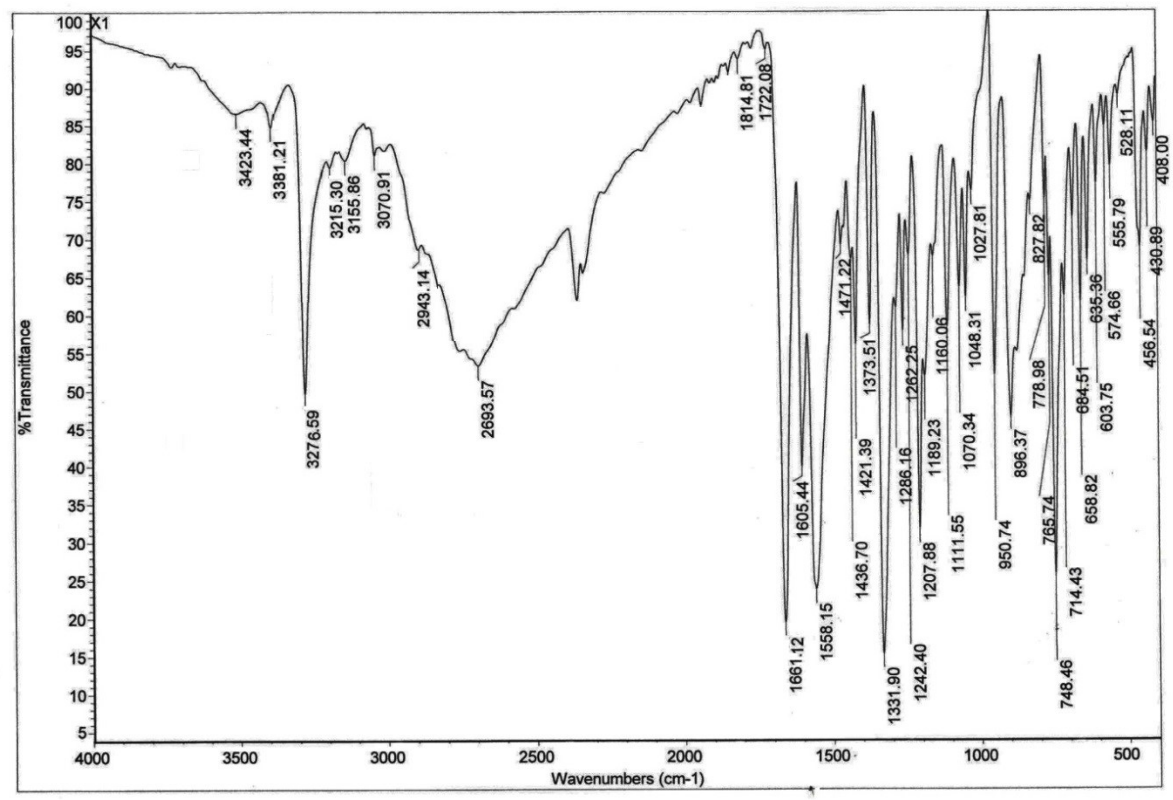

Figure S16. IR spectrum of compound **3d**

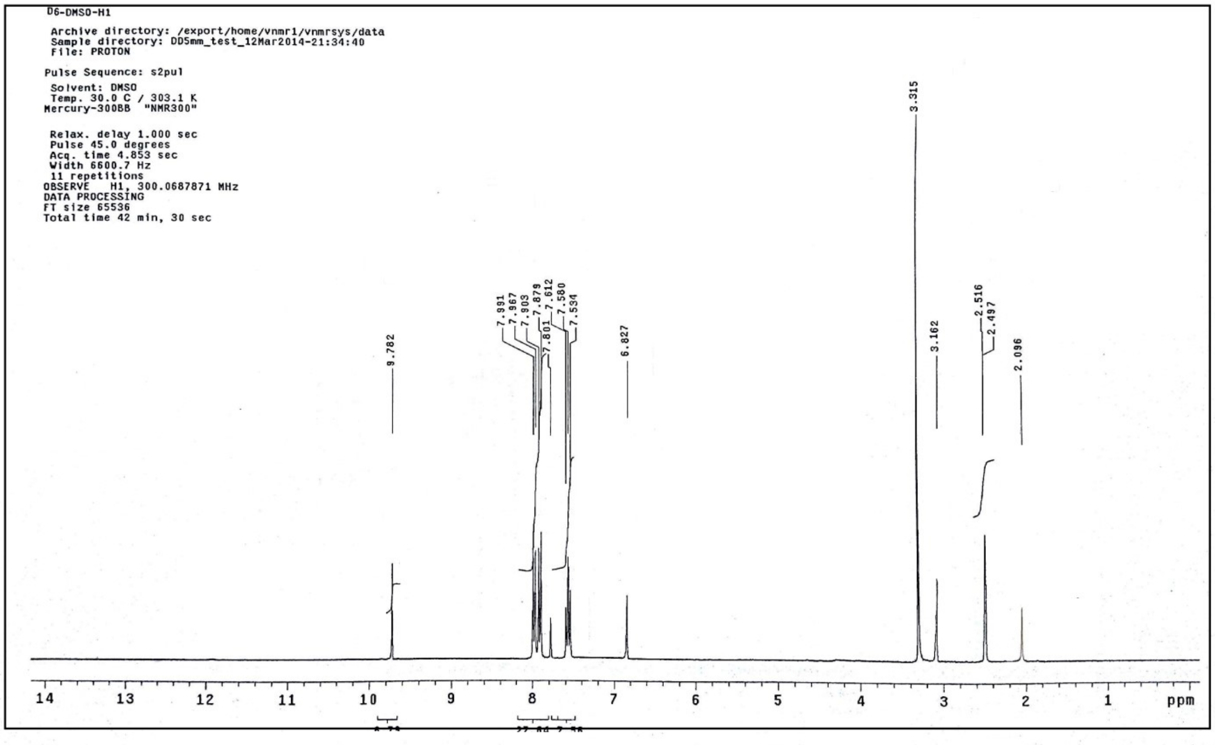

Figure S17. ^{1}H-NMR spectrum of compound **3d**

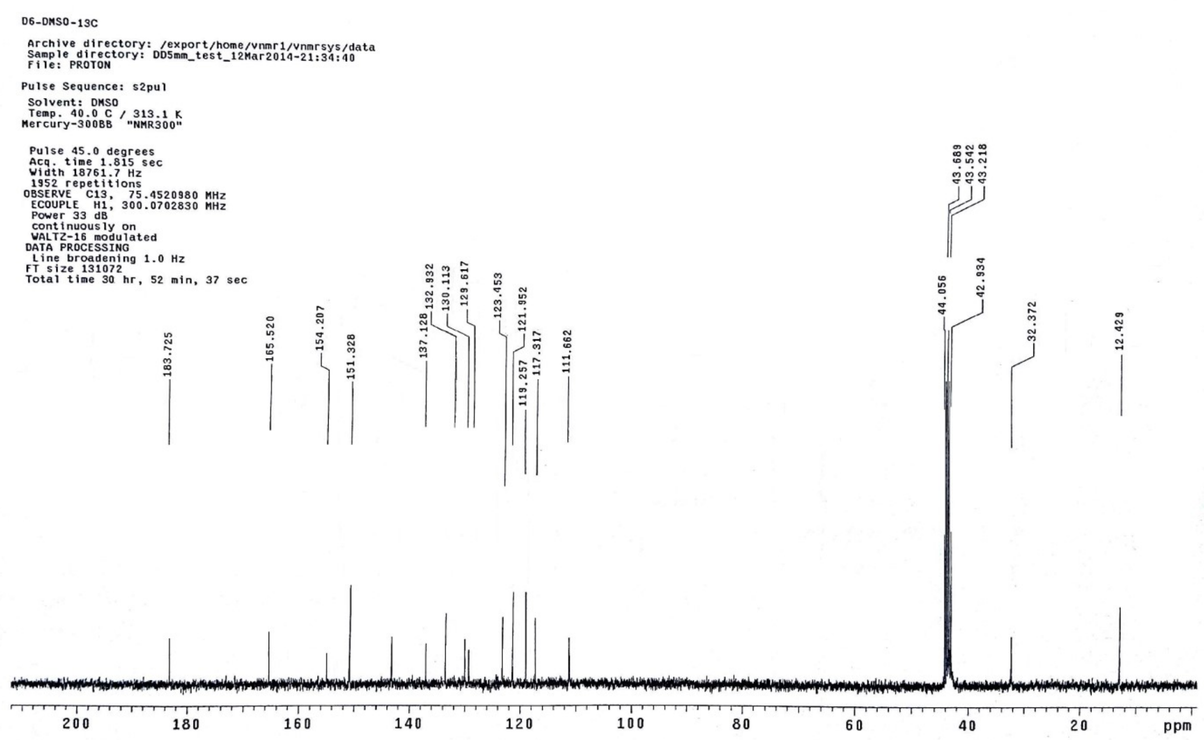

Figure S18. ^{13}C-NMR spectrum of compound **3d**

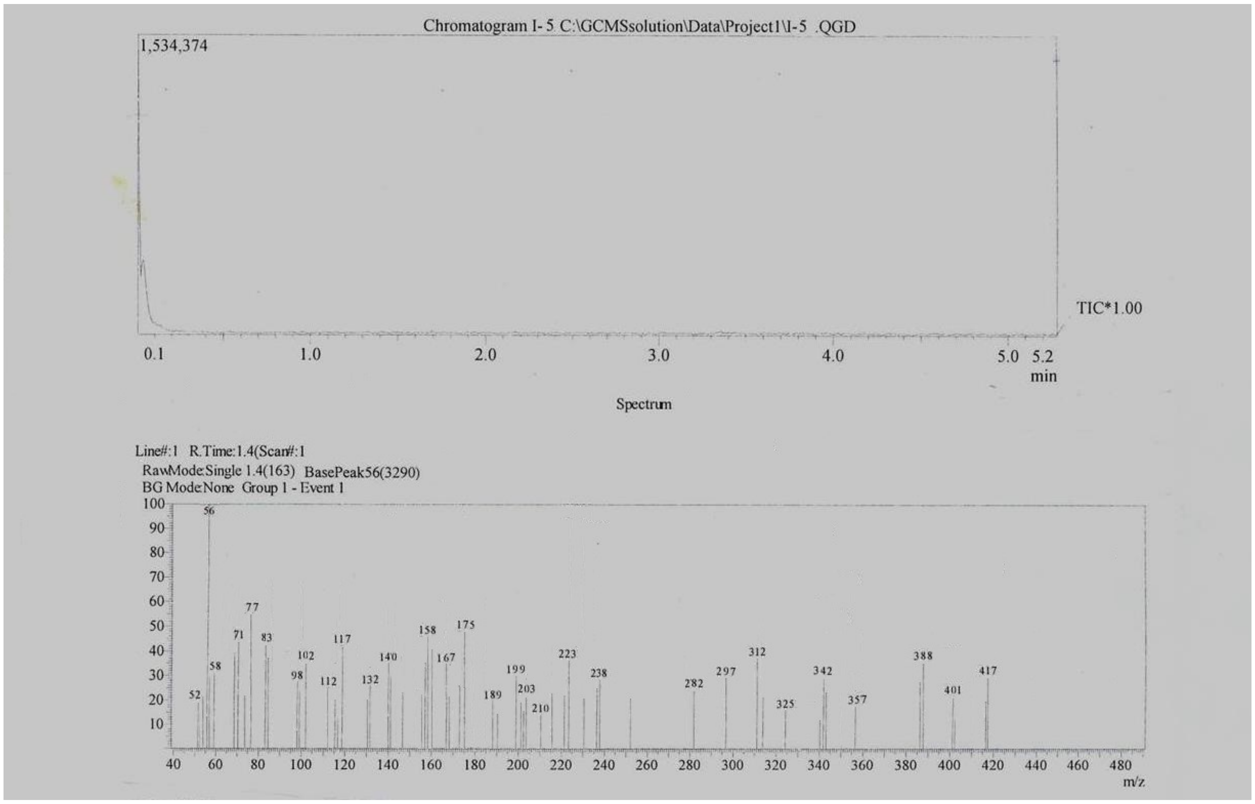

Figure S19. Mass spectrum of compound **3d**

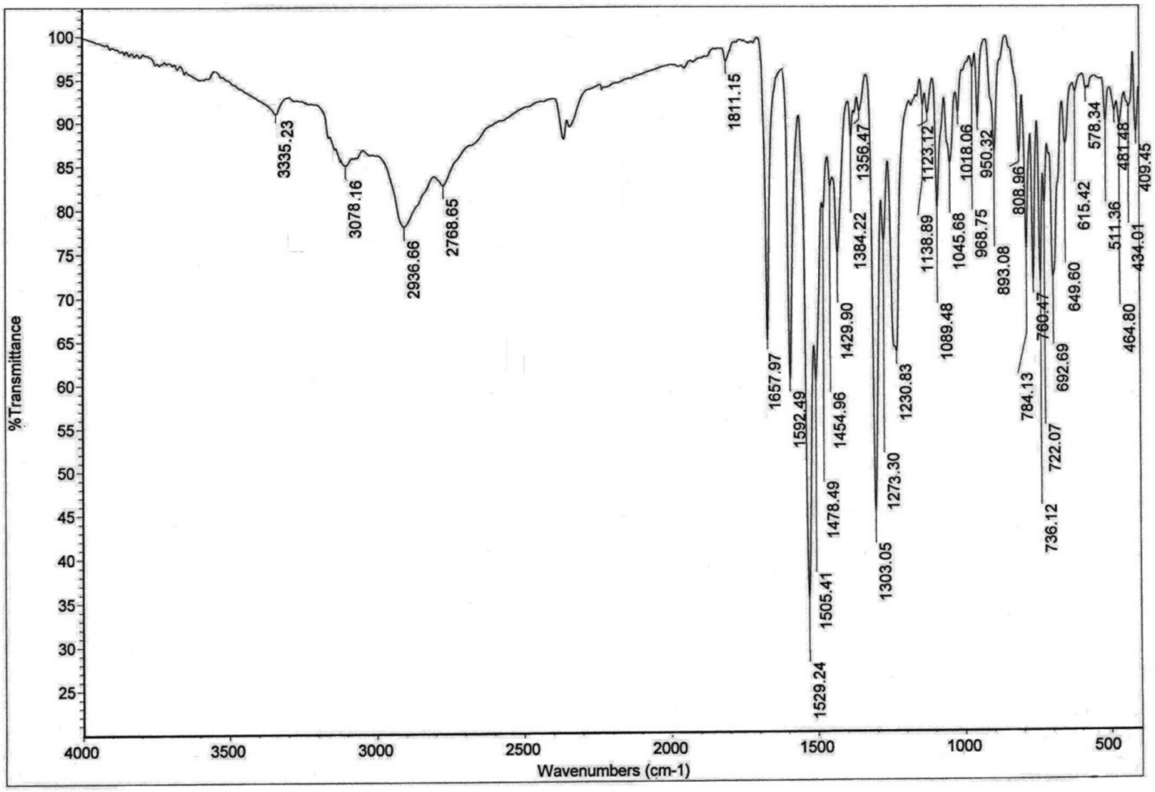

Figure S20. IR spectrum of compound **5a**

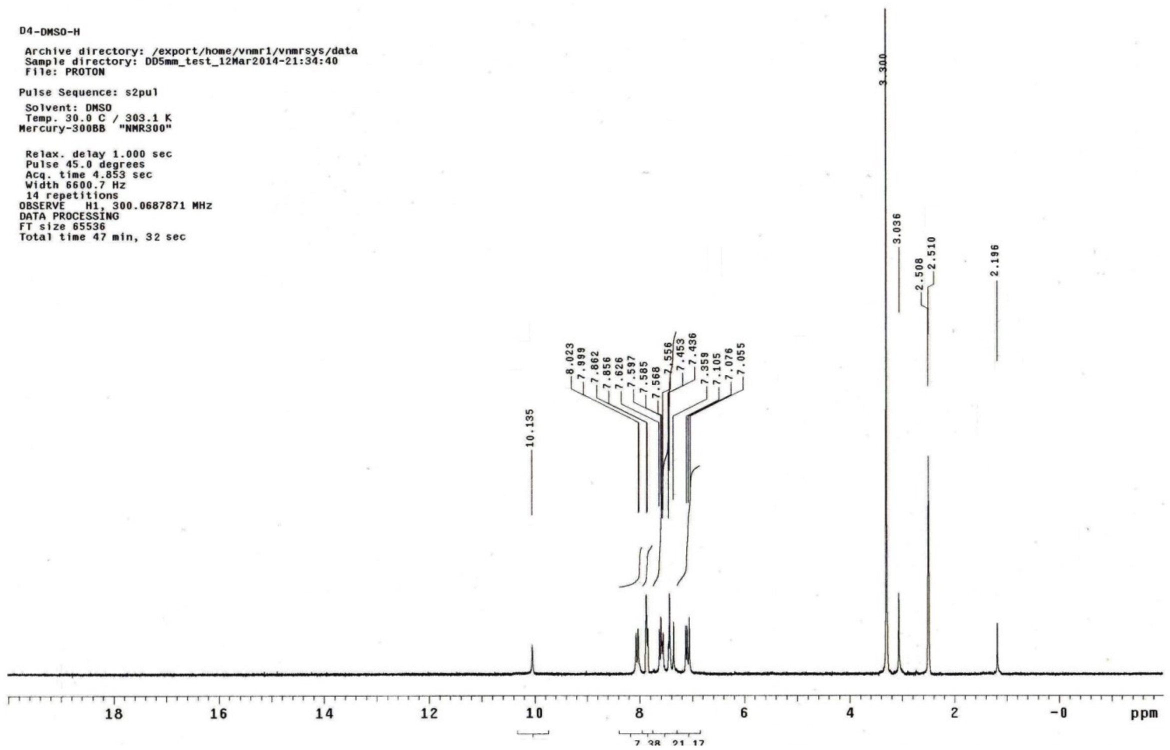

Figure S21. ^{1}H-NMR spectrum of compound **5a**

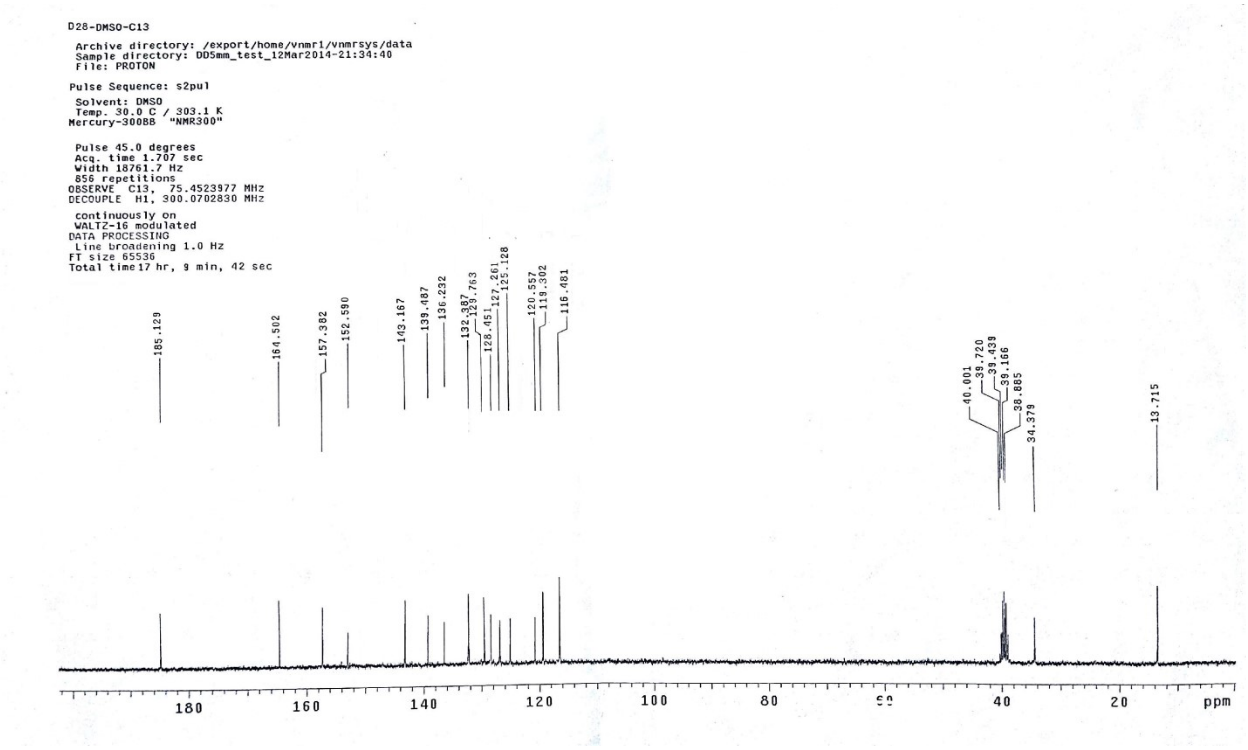

Figure S22. ^{13}C-NMR spectrum of compound **5a**

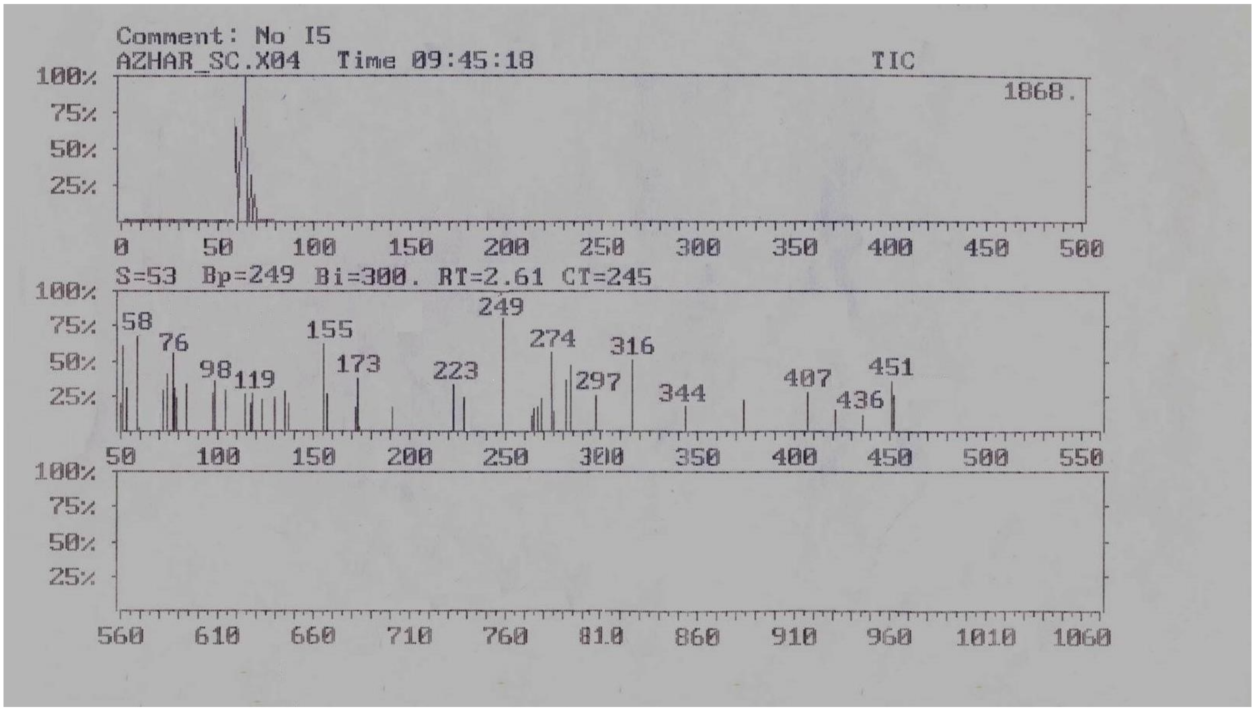

Figure S23. Mass spectrum of compound **5a**

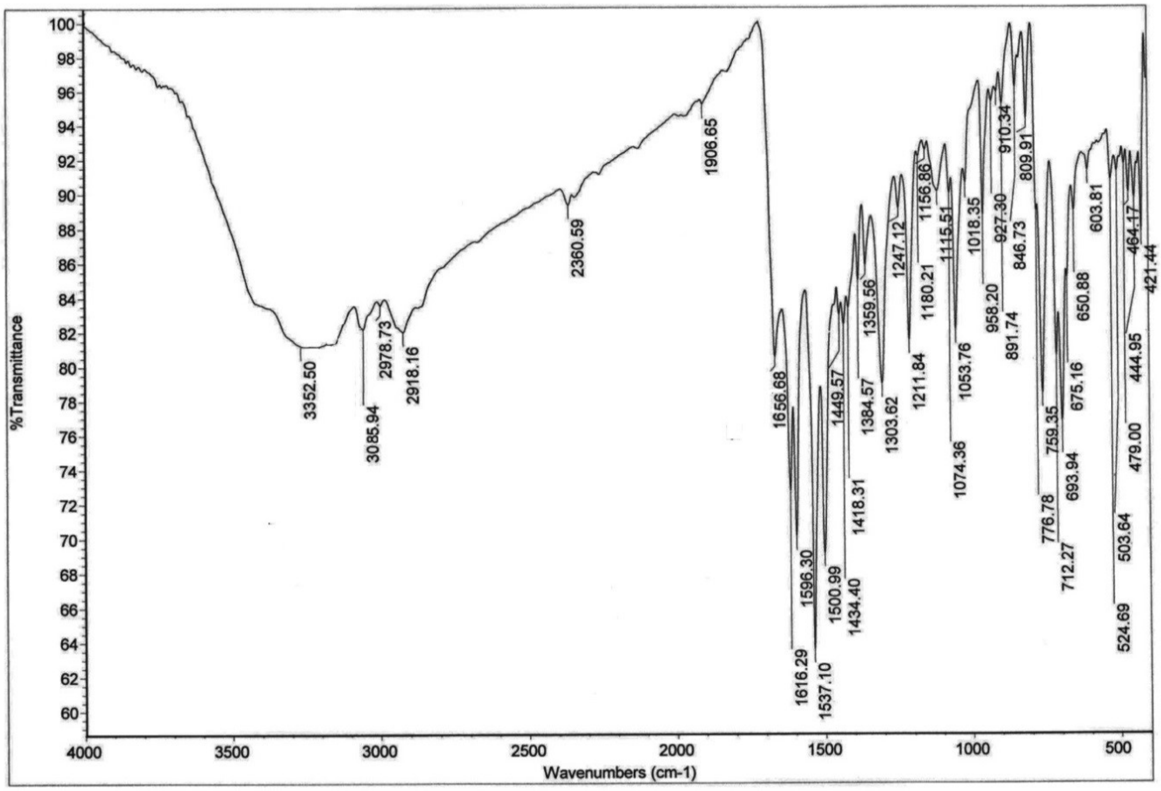

Figure S24. IR spectrum of compound **5b**

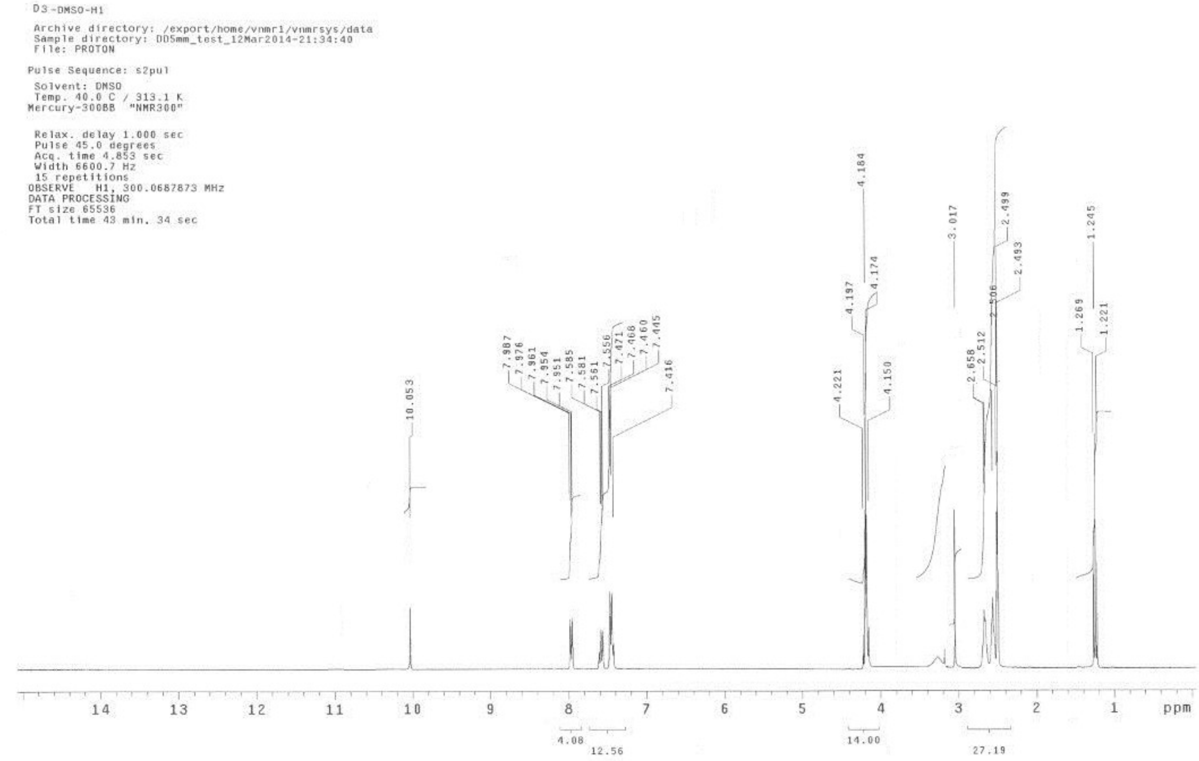

Figure S25. ^{1}H-NMR spectrum of compound **5b**

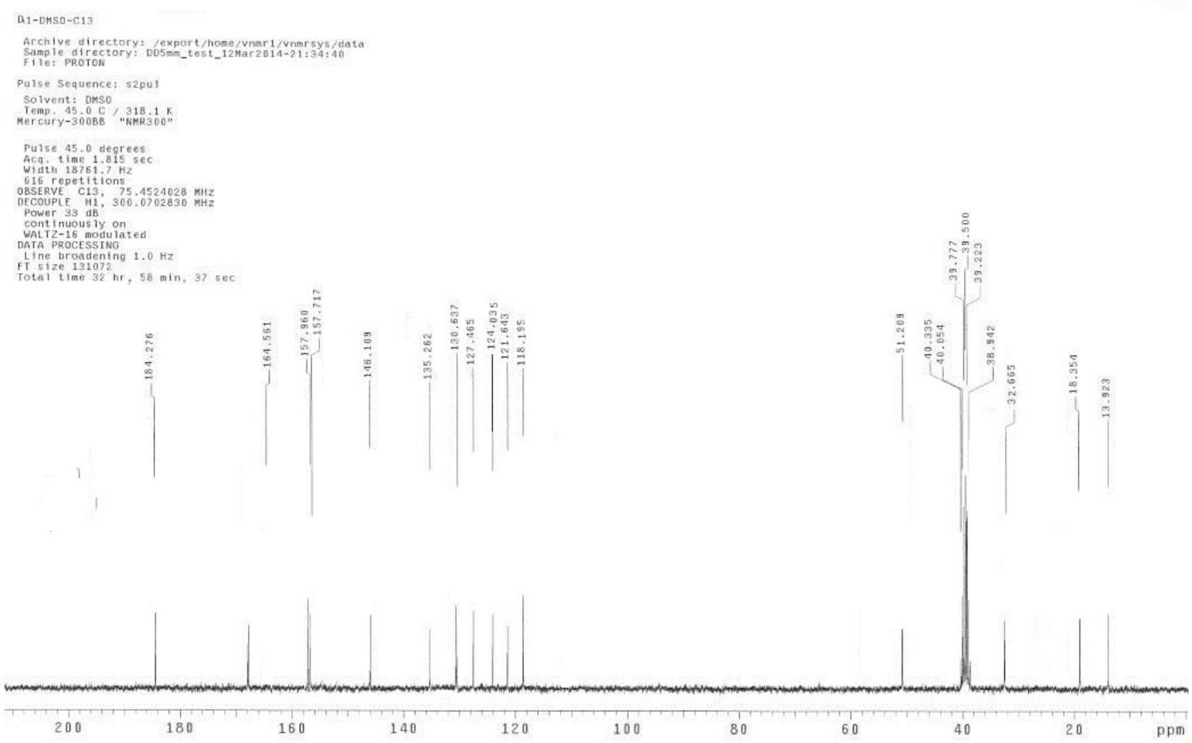

Figure S26. ^{13}C-NMR spectrum of compound **5b**

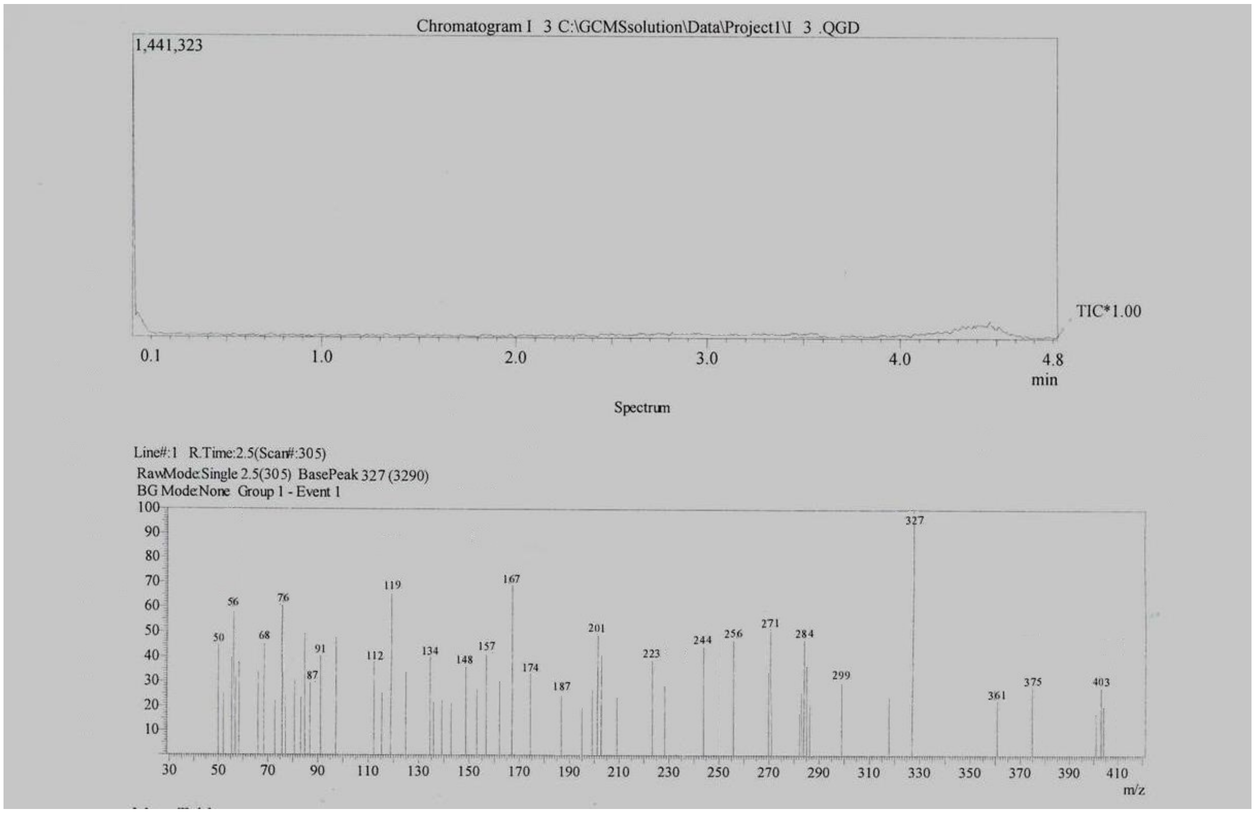

Figure S27. Mass spectrum of compound **5b**

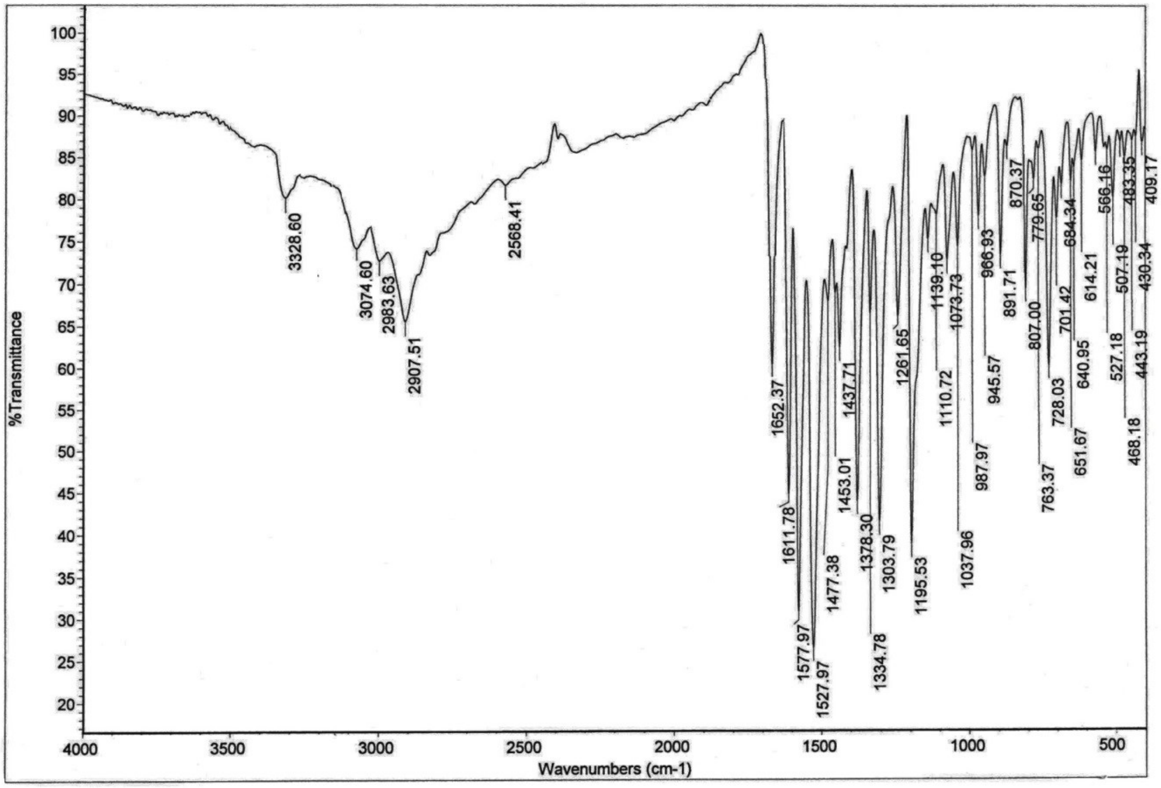

Figure S28. IR spectrum of compound **5c**

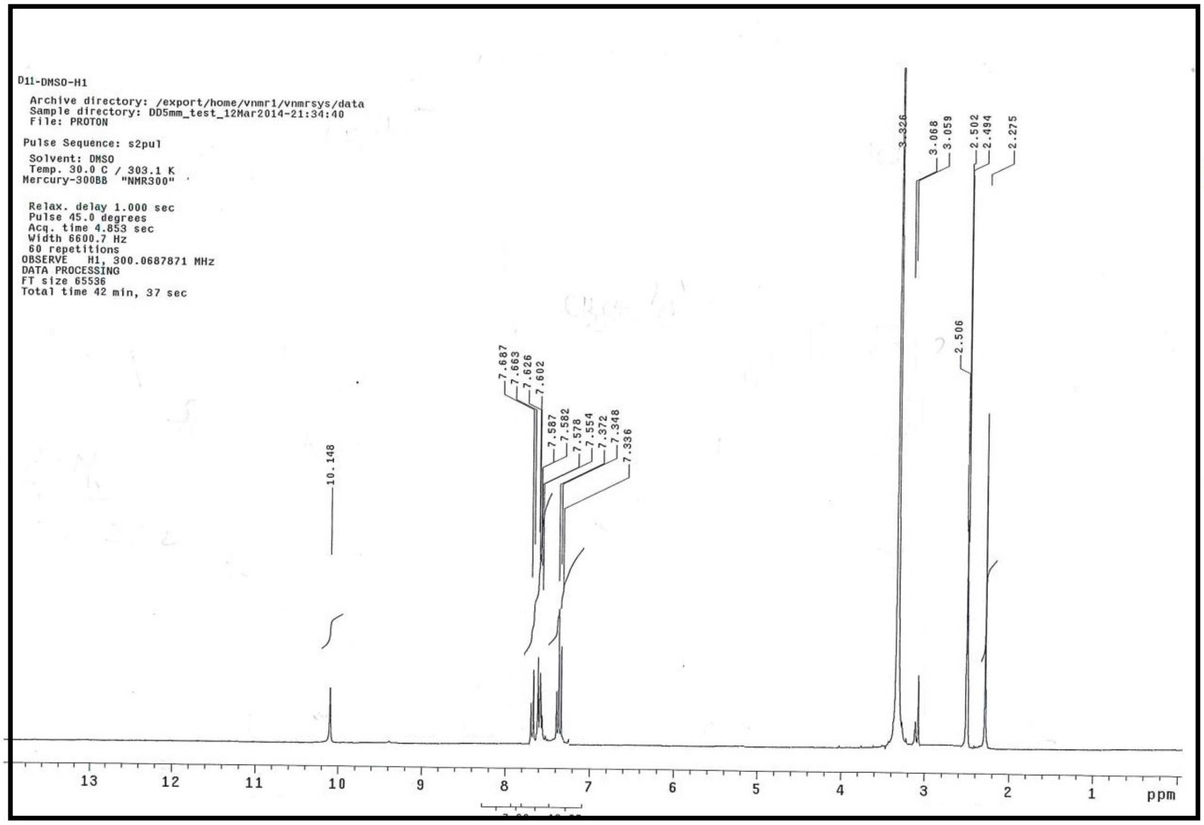

Figure S29. ^{1}H-NMR spectrum of compound **5c**

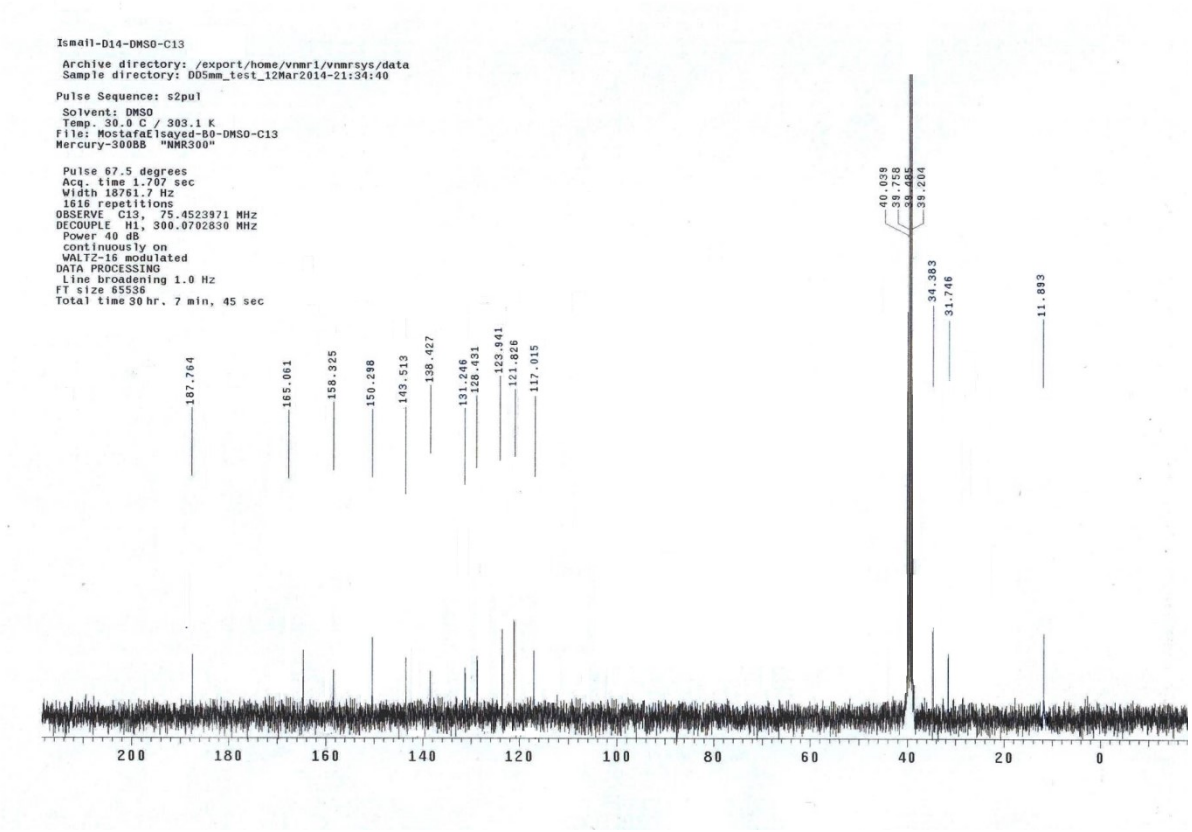

Figure S30. ^{13}C-NMR spectrum of compound **5c**

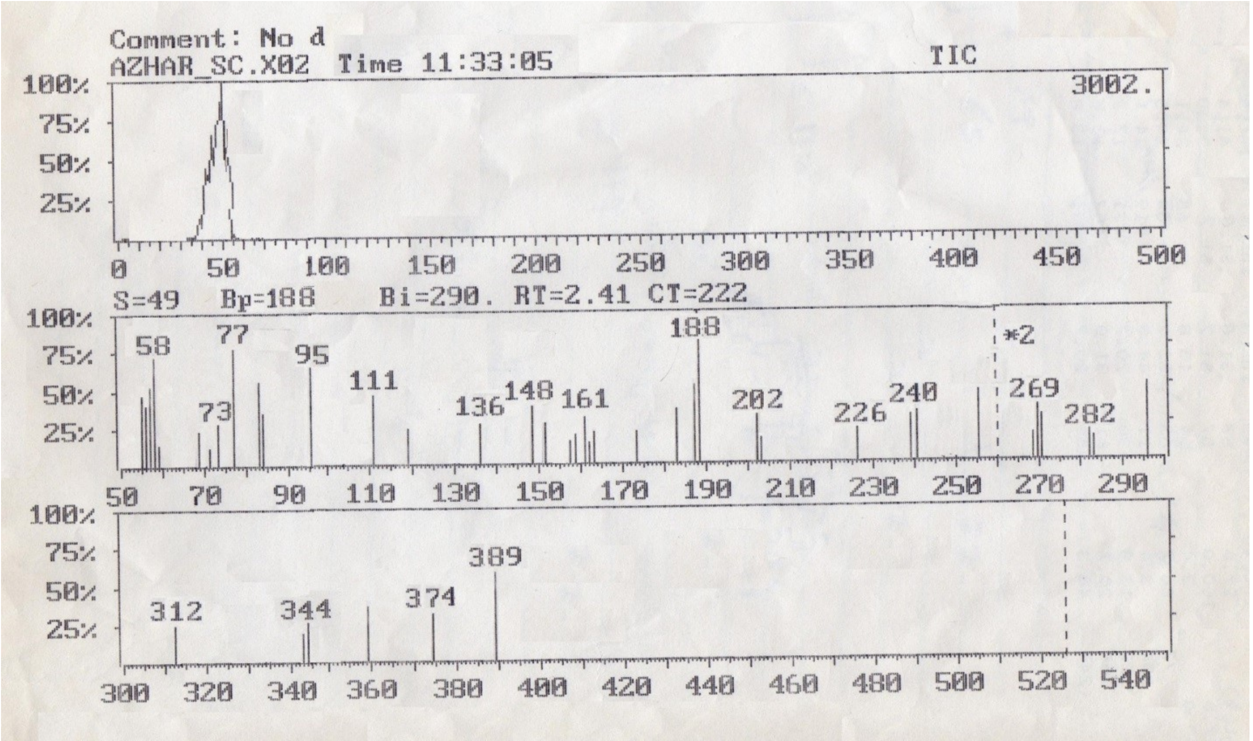

Figure S31. Mass spectrum of compound **5c**

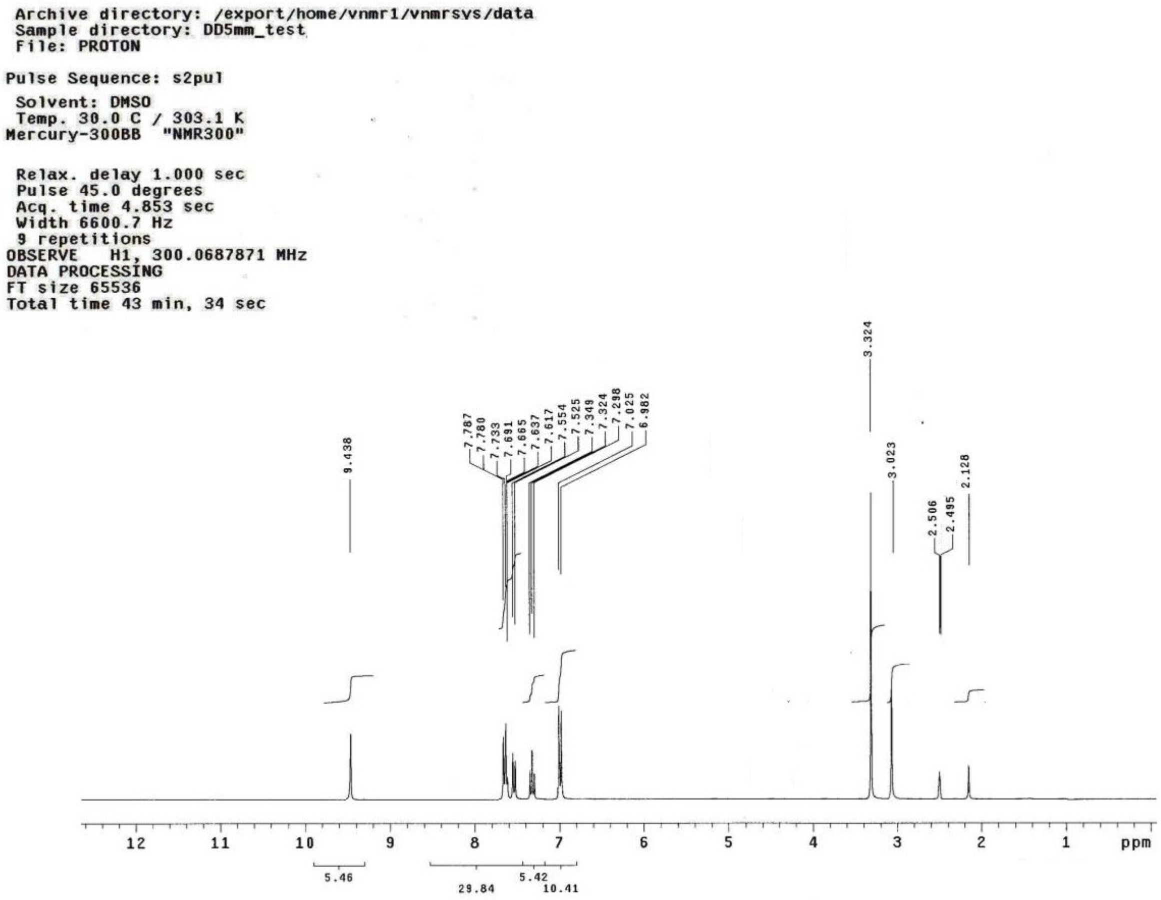

Figure S32. ^{1}H-NMR spectrum of compound **5d**

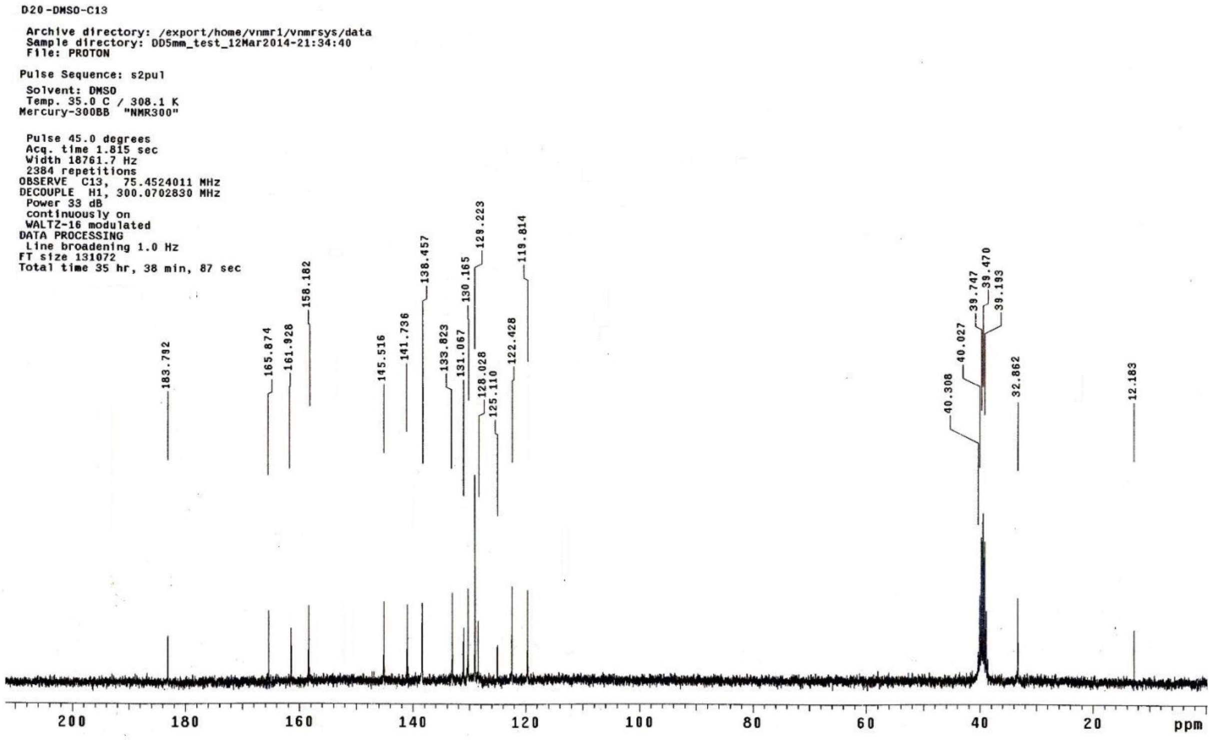

Figure S33. ^{13}C-NMR spectrum of compound **5d**

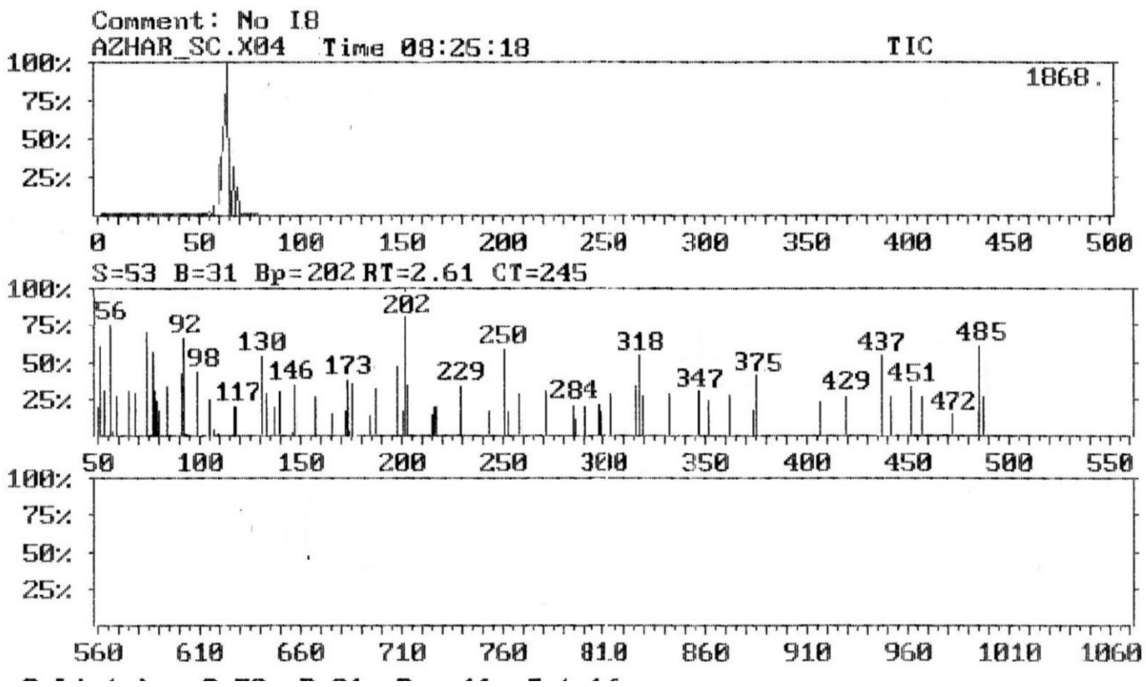

Figure S34. Mass spectrum of compound **5d**
